# Molecular fingerprints of nuclear genome and mitochondrial genome for early diagnosis of lung adenocarcinoma

**DOI:** 10.1186/s12967-023-04099-2

**Published:** 2023-04-10

**Authors:** Yichun Xu, Yong Yang, Yichao Wang, Jun Su, Tianlong Chan, Jiajing Zhou, Yi Gong, Ke Wang, Yifeng Gu, Congmeng Zhang, Guanjin Wu, Ling Bi, Xiong Qin, Junsong Han

**Affiliations:** 1 National Engineering Research Center for Biochip at Shanghai and Shanghai Biochip Limited Corporation, No.151, Libing Road, Shanghai, 201203 China; 2 Department of Thoracic Surgery, Shanghai Pulmonary Hospital, No.241, Huaihai West Road, Shanghai, China; 3 Department of Oncology, Yueyang Hospital of Integrated Traditional Chinese and Western Medicine, Shanghai University of Traditional Chinese Medicine, No.110, Ganhe Road, Shanghai, China; 4 Department of Pathology, Shanghai Tongji Hospital, Tongji Hospital Affiliated to Tongji University, Shanghai, China; 5 Institute of Interdisciplinary Integrative Medicine Research, Shanghai University of Traditional Chinese Medicine, Shanghai, China; 6 Acupuncture Anesthesia Clinical Research Institute, Yueyang Hospital of Integrated Traditional Chinese and Western Medicine, Shanghai University of Traditional Chinese Medicine, Shanghai, China

**Keywords:** Lung adenocarcinoma, Cell-free mtDNA, Mutations, Diagnosis

## Abstract

**Background:**

Lung adenocarcinoma (LUAD) is the most prevalent subtype of lung cancer with high morbidity and mortality rates. Due to the heterogeneity of LUAD, its characteristics remain poorly understood. Exploring the clinical and molecular characteristics of LUAD is challenging but vital for early diagnosis.

**Methods:**

This observational and validation study enrolled 80 patients and 13 healthy controls. Nuclear and mtDNA-captured sequencings were performed.

**Results:**

This study identified a spectrum of nuclear and mitochondrial genome mutations in early-stage lung adenocarcinoma and explored their association with diagnosis. The correlation coefficient for somatic mutations in cfDNA and patient-matched tumor tissues was high in nuclear and mitochondrial genomes. The mutation number of highly mutated genes was evaluated, and the Least Absolute Shrinkage and Selection Operator (LASSO) established a diagnostic model. Receiver operating characteristic (ROC) curve analysis explored the diagnostic ability of the two panels. All models were verified in the testing cohort, and the mtDNA panel demonstrated excellent performance. This study identified somatic mutations in the nuclear and mitochondrial genomes, and detecting mutations in cfDNA displayed good diagnostic performance for early-stage LUAD. Moreover, detecting somatic mutations in the mitochondria may be a better tool for diagnosing early-stage LUAD.

**Conclusions:**

This study identified specific and sensitive diagnostic biomarkers for early-stage LUAD by focusing on nuclear and mitochondrial genome mutations. This also further developed an early-stage LUAD-specific mutation gene panel for clinical utility. This study established a foundation for further investigation of LUAD molecular pathogenesis.

**Supplementary Information:**

The online version contains supplementary material available at 10.1186/s12967-023-04099-2.

## Introduction

Lung cancer is a major cause of mortality worldwide, responsible for 1,796,000 deaths in 2020, and lung adenocarcinoma (LUAD) is the most common subtype [1]. Smoking is usually considered as the main cause of lung cancer. However, LUAD is more likely to occur in non-smoking women and youngsters [2, 3]. Complete surgical resection is the most effective therapy for LUAD. However, many patients are diagnosed at the metastasis or advanced stages of cancer progression. A spectrum of nuclear and mitochondrial genome mutations can be identified in early-stage lung adenocarcinoma, and their association with diagnosis has been explored [4]. However, late diagnosis and the high mutational burden encountered in lung cancer remain a problem [5]. Therefore, it is essential to improve LUAD’s early diagnosis rate.

Recently, high-throughput sequencing and microarray technologies have been used in biomarker research for cancer diagnosis and prognosis [6, 7]. lncRNAs, miRNAs, and mRNAs expressions were all associated with LUAD occurrence. These include DiGeorge syndrome critical region gene 5 (DGCR5), kinesin family member 20A (KIF20A), C-type lectin domain family 10, member A (CLEC10A), and has-miR-29c [8–11]. DNA methylation biomarkers also contribute to lung cancer diagnosis. Furthermore, epithelial gene cadherin 1 (Cdh1) and epithelial cell adhesion molecule (EpCAM) are key features of the epithelial–mesenchymal transition (EMT) process that are significantly hypermethylated in lung cancer [12]. The most frequent LUAD-driving genes are epidermal growth factor receptor (EGFR), KRAS proto-oncogene, GTPase (KRAS), B-Raf proto-oncogene, serine/threonine kinase (BRAF), and erb-b2 receptor tyrosine kinase 2 (ERBB 2). These genes are directly correlated with the diagnosis, treatment efficacy, and prognosis of LUAD [13–15].

Liquid biopsy is a non-invasive tool for cancer diagnosis, monitoring, and treatment decisions [16]. Circulating cell-free DNA (cfDNA) or circulating tumor cells (CTCs) in plasma or other body fluids are usually used in liquid biopsy assays [17]. Somatic alterations are detected in EGFR, tumor protein p53 (TP53), and BRCA2 DNA repair associated (BRCA2) in plasma or serum ctDNA. These alterations are associated with diagnosis, therapy resistance, and response [18–21]. Most of these studies focused on nuclear-origin cfDNA, but the amount of tumor-derived cfDNA of nuclear origin is extremely low in many early-stage cancers [22, 23]. mtDNA has a higher copy number than nuclear DNA (nDNA) and is susceptible to mutations [24]. Increasing mtDNA copy number may compensate for mtDNA damage or dysfunction [25]. An elevated mtDNA copy number in the blood is linked to an increased risk of several malignancies, including non-Hodgkin lymphoma [26], colorectal cancer [27], lung cancer [28], and pancreatic cancer [29].

The current understanding of circulating cell-free mitochondrial DNA has great potential as a novel tumor biomarker [30]. Our previous study found that the content and variants of circulating mitochondrially encoded NADH dehydrogenase 1 (MT-ND1) may become a versatile tool for diagnosing and monitoring colorectal cancer [27]. However, no systematic comparisons between liquid and solid biopsies of the mitochondrial genome have been performed.

The major genomic alterations of 131 Stage IA LUAD were systematically examined using The Cancer Genome Atlas (TCGA) database. This study conducted a whole exome sequencing (WES) profile and captured-based mitochondrial sequencing diagnosed with early-stage Stage IA LUAD, followed by bioinformatic approaches to identify a panel of key genes in the genome and mitochondrial genome in plasma of LUAD. A novel mutational signature for early diagnosis in the genome and mitochondrial genome in the plasma of LUAD was proposed. This research demonstrated that cell-free mtDNA from plasma is a potential biomarker for early-stage LUAD diagnosis.

## Materials and methods

### TCGA data download and analysis

The Cancer Genome Atlas (TCGA) is a cancer genomics program that provides publicly available data that contributes to cancer studies (https://www.cancer.gov/about-nci/organization/ccg/research/structural-genomics/tcga). WES profiles and associated clinicopathological data of Stage IA LUAD patients were retrieved on 1st March 2020 from the TCGA database. The analysis included 131 pairs of LUAD tissue samples and adjacent normal tissue samples. Somatic mutation data were identified using four different somatic mutation-calling algorithms (VarScan, SomaticSniper, MuTect, and MuSE) of the ‘maftools’ in the R package.

### Patients and study design

This research obtained primary Stage IA LUAD tissues, their adjacent tissues, and blood from 80 patients not priorly treated with chemotherapy or radiotherapy. All 80 patients and 13 healthy controls provided informed written consent. All experiments followed the relevant guidelines and regulations at Shanghai Pulmonary Hospital. The approval number for the present study was 2020-038.

For comparative purposes, the study included.

- TCGA cohort’s WES profile (131 Stage IA tumor tissues and conditionally normal adjacent tissues from the TCGA database).
- WES data from 15 pairs of primary Stage IA LUAD tumor tissues and conditionally normal adjacent tissues.
- Targeted sequencing data from 43 pairs of primary Stage IA LUAD tumor tissues and conditionally normal adjacent tissues.
- Mitochondrial sequencing data from 43 pairs of primary Stage IA LUAD tumor tissues and conditionally normal adjacent tissues.
- Targeted sequencing data from plasma samples of 25 Stage IA LUAD patients.
- Mitochondrial sequencing data from plasma samples of 20 Stage IA LUAD patients.
- Targeted sequencing data from plasma samples of six healthy individuals for training
- Mitochondrial sequencing data from plasma samples of six healthy individuals for training.
- Targeted sequencing data from plasma samples of seven Stage IA LUAD patients for testing.
- Mitochondrial sequencing data from plasma samples of seven Stage IA LUAD patients for testing.
- Targeted sequencing data from plasma samples of seven healthy individuals for testing.
- Mitochondrial sequencing data from plasma samples of seven healthy individuals for testing.

### Sample collection

The Tissue Genomic DNA Isolation Kit (Shanghai Biochip Inc., China) was used following the manufacturer’s instructions for tissue DNA extraction. Blood samples were collected in tubes with 0.5 M EDTA solution. The tubes were centrifuged at 2000×*g* for 10 min at 4 °C to collect the plasma. The plasma samples were then centrifuged again at 16,000×*g* for 10 min at 4 °C. Plasma samples were collected and stored at − 80 °C. The QIAamp Circulating Nucleic Acid Kit (QIAGEN, Germany) was used for cfDNA extraction. The Qubit dsDNA HS Assay Kit (Life Technologies) and Agilent 4200 Bioanalyzer determined DNA concentrations and cfDNA quality, respectively.

### Library preparation, target capture, and next-generation sequencing

The Twist Human Core Exome Kit (Twist Bioscience, San Francisco, CA, USA) performed WES for exome-targeted library enrichment. This kit was about 56.6 M covering the consensus coding sequence (CCDS) region, non-protein coding exonic region, and the region surrounding the transcription start site. The exome capture kit covered approximately 99.841% of the reference gene CDS region. Exomes were sequenced on an Illumina NovaSeq (Illumina) according to the manufacturer’s instructions.

The mtDNA sequence was sequenced using a capture-based mtDNA deep-sequencing approach. Dynagen Bioscience provided QuarXeq Mitochondrial Probes (Y1035A). The custom panel was approximately 1.5 M, covering 115 selected genes synthesized by Dynegen Bioscience. Then, 500 ng genomic DNA and 30 ng cfDNA were used for library construction, and Dynegen Kits were used. Library quantification was performed using an Agilent 4200 Bioanalyzer before and after PCR amplification. Both panels were sequenced on an Illumina NovaSeq (Illumina) according to the manufacturer’s instructions.

### Data analysis

The human reference genome (hg38) was downloaded from the UCSC genome table browser (http://genome.ucsc.edu/). The revised Cambridge Reference Sequence (rCRS) provided the mitochondrial genome (AC: NC_012920). Sequencing data for nuclear and mitochondrial genome were obtained following standard methods prior to the experiment procedures. Fastp v0.21.0 performed quality checks for the sequenced reads. Read mapping was aligned to the reference genome using BWA version 0.7.17 [31], and duplicated reads were removed using Sambamba v0.6.8 (http://lomereiter.github.io/sambamba). GATK Mutect2 (Genome Analysis Toolkit) (https://www.broadinstitute.org/gatk) called up the somatic single-nucleotide variant (SNV) and indel mutations with a minimum of five mutant allele read. GATK Mutect2 was used for mtDNA in mitochondrial mode to call mutations, and GATK FilterMutectCalls filtered the sequenced data. Variants in the nuclear and mitochondrial genomes were annotated with Annovar and GATK Funcotator, respectively. Tumor mutational burden (TMB) (mutations per Mb) was calculated by considering the number of nuclear genomic positions in the coding region with sufficient coverage to detect a mutation with the same variant allele frequencies (VAF). TMB for mtDNA was calculated by considering the number of mitochondrial genomic positions in all regions with sufficient coverage to detect mutations with the same VAF.

### Statistical analysis

The R v.4.0.3 environment (https://www.r-project.org/) and RStudio v1.1 (https://www.rstudio.com/) performed bioinformatic analysis using the packages of ggplot2 (v3.3.5), maftools (v2.4.12), pROC (v1.18.0), and Circlize (v0.4.13). Wilcoxon signed-rank test compared TMB between groups. Fisher exact test and Chi-square tests were performed to evaluate the significance of mutation hotspot numbers between the different groups. Statistical significance was set at *p* < 0.05.

## Results

### Study cohort’s clinical characteristics

This observational and validation study enrolled 80 patients and 13 healthy controls. Clinical characteristics of the study cohort included age, gender, pathology, TNM stage, and smoking status (Table 1).

**Table 1:** Clinical characteristics of the study cohort (n = 93)

	LUAD (n = 80)	Healthy individuals (n = 13)
Age (years), median (range)	60.31 (32–77)	48(29–66)
< 60, n (%)	41 (51.25)	7 (53.85)
≥ 60, n (%)	39 (48.75)	6 (46.15)
Gender, n (%)		
Male	22 (27.50)	5 (38.46)
Female	58 (72.50)	8 (61.54)
Stage		
TIA1, n (%)	15 (18.75)	–
TIA2, n (%)	38 (47.50)	–
TIA3, n (%)	27 (33.75)	–
Infiltration		
MIA, n (%)	17 (21.25)	–
IA, n (%)	63 (78.75)	–

### Genomic alterations in early-stage LUAD of TCGA database

**Fig. 1:**
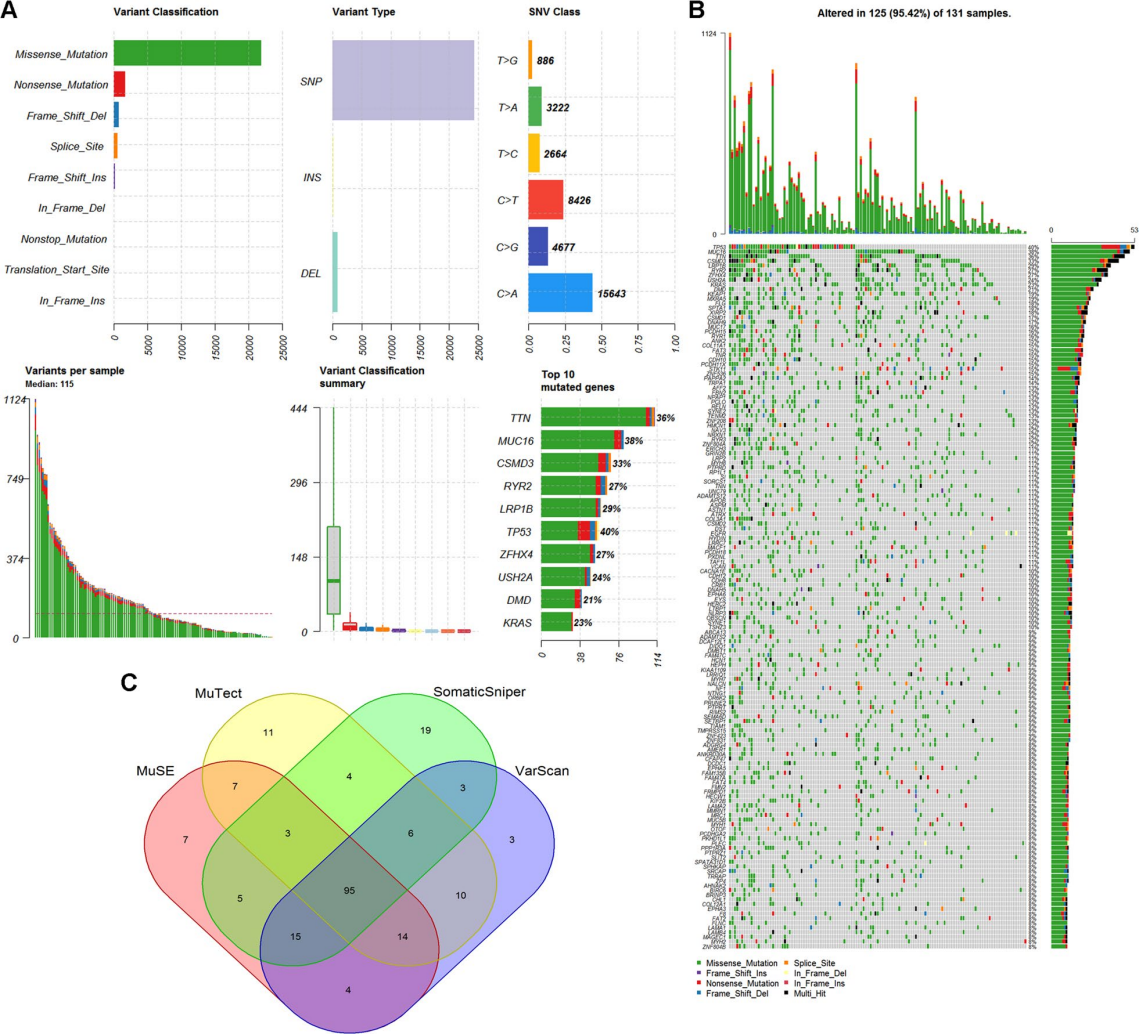
TGCA early-stage LUAD mutation cohort. **A** Overview of TGCA Stage IA LUAD mutation cohort analyzed with the VarsSan tool. **B** Waterfall of the top 150 mutated genes in the TCGA Stage IA LUAD cohort was analyzed with the tool of VarScan. **C** Representative Venn diagrams of mutated gene numbers called by VarsSan, SomaticSniper, MuTect, and MuSE

This study analyzed gene mutations across 131 samples of early-stage LUAD in the TCGA database to systematically characterize genomic alterations that occur in early-stage LUAD. Varscan, Somaticsniper, and Muse tools were employed to construct a mutant gene profile for early-stage LUAD using the WES profile from TCGA. Genomic mutation information was analyzed by VarScan (Fig. 1A, B), SomaticSniper (Additional file 1: Figure S1A, B), MuTect (Additional file 1: Figure S2A, B), and MuSE (Additional file 1: Figure S3A, B).

VarScan identified 12,575 mutated genes with a median of 115 mutated genes per sample (Fig. 1A). The top five mutated genes were titin (TTN) (35.8% of patients, 47/131), mucin 16, cell surface-associated (MUC16) (38.2%, 50/131), CUB and Sushi multiple domains 3 (CSMD3) (32.8%, 43/131), ryanodine receptor 2 (RYR2) (26.7%, 35/131), and LDL receptor-related protein 1B (LRP1B) (29.0%, 38/131) (Fig. 1B). SomaticSniper identified 10,554 mutated genes, with a median of 78.5 mutated genes per sample (Additional file 1: Figure S1A). The top five mutated genes were TTN (31.3% of patients, 41/131), MUC16 (29.8%, 39/141), CSMD3 (26.7%, 35/131), RYR2 (22.1%, 29/131), and TP53 (32.1%, 42/131) (Additional file 1: Figure S1B). MuTect identified 11,049 mutated genes with a median of 151 per sample (Additional file 1: Figure S2A). The top five mutated genes were TTN (41.2% of patients, 54/131), MUC16 (38.9%, 51/131), CSMD3 (35.8%, 47/131), RYR2 (32.1%, 42/131), and LRP1B (35.1%, 46/131) (Additional file 1: Figure S2B). MuSE identified 12,541 mutated genes with a median of 117 mutated genes per sample (Additional file 1: Figure S3A). The top five mutated genes were TTN (36.6% of patients, 48/131), MUC16 (35.1%, 46/131), CSMD3 (32.1%, 42/131), RYR2 (28.2%, 37/131), and LRP1B (29.8%, 39/131) (Additional file 1: Figure S3B).

The intersection of mutant genes analyzed by VarScan, SomaticSniper, MuTect, and MuSE revealed 95 co-mutant genes (Fig. 1C). The most frequent identified alterations occurred in TTN, MUC16, TP53, CSMD3, LRP1B, RYR2, zinc finger homeobox 4 (ZFHX4), usherin (USH2A), filaggrin (FLG), and dystrophin (DMD). TTN, MUC16, CSMD3, RYR2, LRP1B, TP53, and ZFHX4 were the top ten mutated genes analyzed by the four tools. Missense mutation was the leading variant classification. The non-synonymous mutation rate was significantly lower than the synonymous mutation rate. The other identified mutational signature was characterized by a higher frequency of C>A transitions, comprising more than 40% of the single nucleotides.

### Somatic genomic alterations analyzed by WES

**Fig. 2:**
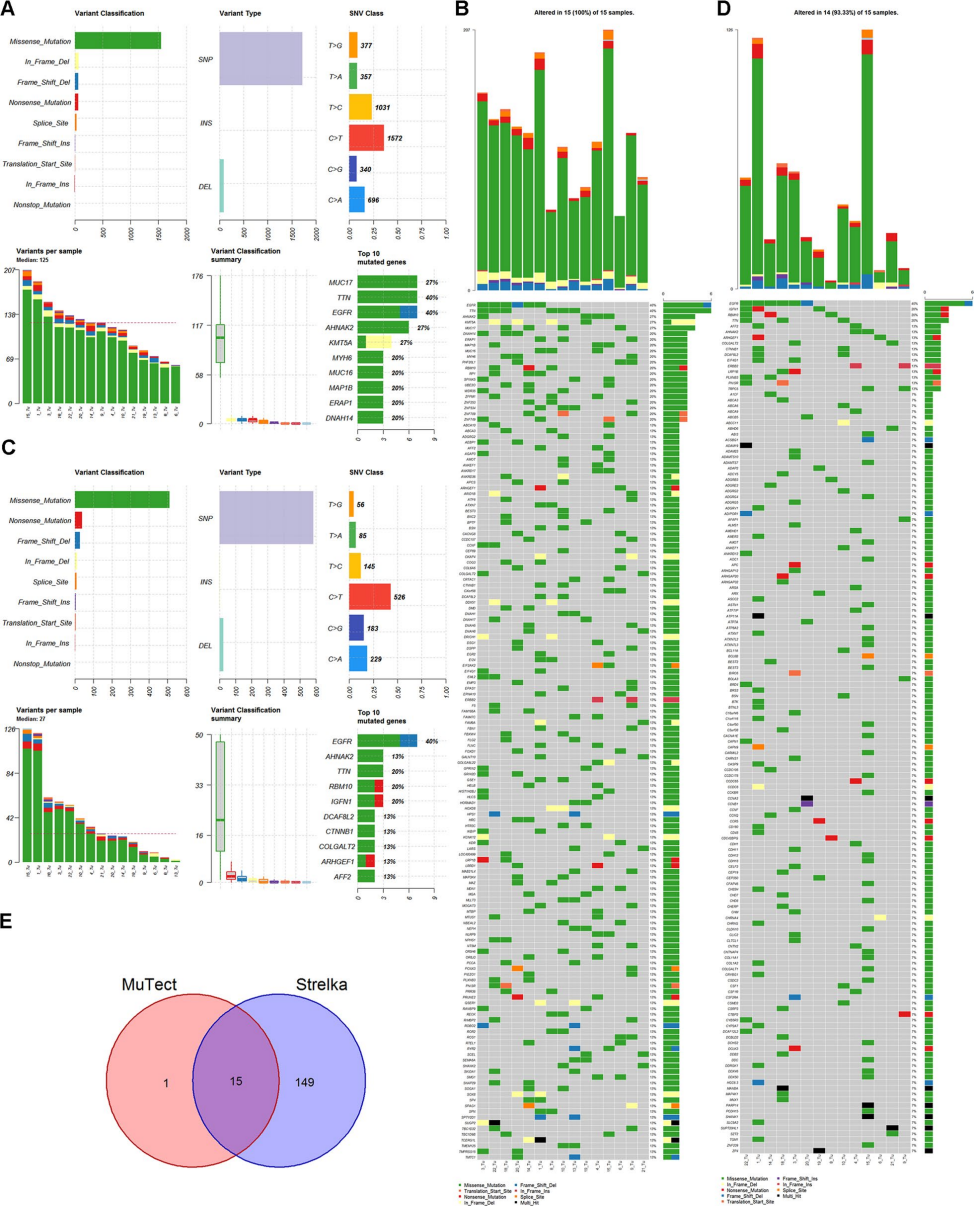
Stage IA LUAD mutation cohort. **A** Overview of TGCA Stage IA LUAD mutation cohort analyzed with the Strelka tool. **B** Waterfall of the top 150 mutated genes in the Stage IA LUAD cohort was analyzed with the tool of Strelka. **C** Overview of Stage IA LUAD cohort mutations analyzed with the tool of GATK. **D** Waterfall of the top 150 mutated genes in the Stage IA LUAD cohort was analyzed with the tool of GATK. **E** Representative Venn diagrams of mutated gene numbers called by MuTect, and Strelka

This study employed Strelka and GATK to identify significantly mutated genes in Stage TIA LUAD. A mutant gene profile was constructed for Stage TIA LUAD using the WES profile from 15 pairs of tumor tissues and their adjacent tissues. The Strelka and GATK methods analyzed genomic mutation information (Fig. 2A–D). Strelka identified 2561 somatic mutations in exons by WES, including 780 synonymous SNVs, 1551 non-synonymous SNVs, and 230 indels. The top 150 mutated genes were defined in 93.3% (14/15) of pairs of tumor tissues and their adjacent tissues, with a median of 125 mutated genes per sample. The most mutated genes were mucin 17, cell surface associated (MUC17) (26.7% of patients), TTN (40.0%), and EGFR (40.0%).

GATK identified 820 somatic mutations by WES, including 215 synonymous SNVs, 512 non-synonymous SNVs, and 93 indels. The top 150 mutated genes were identified in all 15 pairs of tumor tissues and their adjacent tissues, with a median of 27 mutated genes per sample. The most mutated genes contained EGFR (40.0% of patients), AHNAK nucleoprotein 2 (AHNAK2) (40.0%), and TTN (20.0%).

The research focused on mutant genes with at least one somatic mutation in at least two samples and found that 15 mutant genes were screened by the two tools (Fig. 2E). They were EGFR, RNA binding motif protein 10 (RBM10), TTN, AHNAK2, AF4/FMR2 family member 2 (AFF2), Rho guanine nucleotide exchange factor 1 (ARHGEF1), collagen beta (1-*O*)galactosyltransferase 2 (COLGALT2), catenin beta 1 (CTNNB1), DDB1 and CUL4 associated factor 8 like 2 (DCAF8L2), eukaryotic translation initiation factor 4 gamma 1 (EIF4G1), erb-b2 receptor tyrosine kinase 2 (ERBB2), LRP1B, plexin B3 (PLXNB3), PNN-interacting serine and arginine-rich protein (PNISR), and transient receptor potential cation channel’s subfamily C member 5 (TRPC5). Meanwhile, the most common somatic mutations reported previously were also included in the mutant genes of the LUAD cohort’s WES profile, such as EGFR, TTN, CTNNB1, and MUC17 [32, 33]. Missense mutation was the leading variant classification. The other identified signature was characterized by a higher frequency of C>T transitions, comprising more than 35% of all SNVs analyzed by the two tools. In addition, somatic genomic alterations for carcinoma in situ were analyzed, and there were barely any mutations in the tumor tissues compared to their adjacent tissues.

### The custom capture panel information

The custom capture panel combined the analysis results of TCGA and WES databases with the commonly mutated lung cancer genes recommended by the National Comprehensive Cancer Network (NCCN). The NCCN recommends 12 genes, identified relevant variants in multiple solid tumors, and is optimized specifically for lung cancer. These genes include EGFR, ALK receptor tyrosine kinase (ALK), BRAF, KRAS, MET proto-oncogene, receptor tyrosine kinase (MET), ret proto-oncogene (RET), ERBB2, ROS proto-oncogene 1, receptor tyrosine kinase (ROS1), phosphatidylinositol-4,5-bisphosphate 3-kinase catalytic subunit alpha (PIK3CA), NRAS proto-oncogene, GTPase (NRAS), TP53, and mitogen-activated protein kinase kinase 1 (MAP2K1). The capture panel covered 95 driver genes of TCGA primary early-stage LUAD, 15 selected mutations in WES, and 12 recommended NCCN genes. The custom capture panel included 115 genes (Additional file 1: Table S1). The most frequently mutated genes (TTN, TP53, LRP1B, KRAS, AFF2, EGFR, and ERBB2) were detected in two cohorts (TCGA and WES, TCGA and NCCN, and WES and NCCN).

### Mutational landscape of nuclear genome for LUAD tissues

**Fig. 3:**
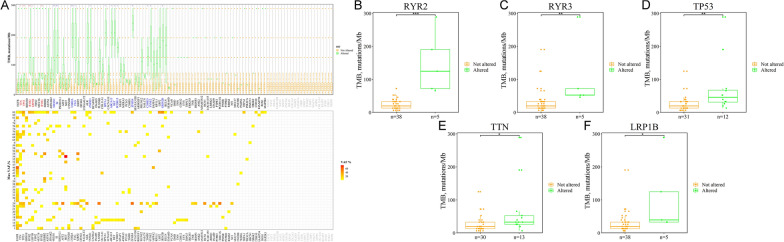
Mutation landscape of nuclear genome in tumor tissues of Stage IA LUAD patients. **A** The mutation landscape of 115 genes in the nuclear genome from 43 tumor tissues of Stage IA LUAD patients. Top: the TMB between the tumor tissues with and without mutations. Bottom: the maximum VAF for each gene in 43 tumor tissues of Stage IA LUAD patients. The TMB between the tumor tissues with and without mutations in **B** RYR2 (*p* < 0.001), **C** RYR3 (*p* < 0.001), **D** TP53 (*p* < 0.01), **E** TTN (*p* < 0.05), and **F** LRP1B (*p* < 0.05) (***p < 0.001; **p < 0.01; *p < 0.05)

This research subjected 43 pairs of tumor tissues and their adjacent tissue samples to targeted sequencing (median depth ×726) of 115 selected genes to identify the landscape of previously detected mutational signatures. The results revealed that all 43 tumor tissues had more than one shared somatic mutation in the custom capture panel. In addition, 95.7% (110/115) genes were identified in these 43 LUAD patients, and 290 somatic mutations were detected in exons by targeted sequencing, including 76 synonymous SNVs, 181 non-synonymous SNVs, and 33 indels. Missense mutation was the leading variant classification. The non-synonymous mutation rate was significantly higher than the synonymous mutation rate. The other identified signature was characterized by a higher frequency of the C:G>T:A transition, comprising 35.0% of all SNVs. The maximum VAFs of somatic mutations in tumor tissues were illustrated in Fig. 3A. The top five mutated genes were EGFR (67.4% of patients, 29/43), TTN (30.2%, 13/43), TP53 (27.9%, 12/43), RBM10 (11.6%, 5/43), and RYR2 (11.6%, 5/43). Mutations occurred in 27.0% (31/115) of genes in the panel of No. 83 Patient, and the patient harbored mutations in genes (EGFR, TTN, TP53, and RYR2). When the tumor tissues had mutations in these genes (RYR2, RYR3, TP53, TTN, and LRP1B), TMB was significantly higher (Fig. 3B–F).

TMB in the tumor tissues with mutated RYR2 (n = 5) was much higher than that in non-mutated RYR2 (n = 38) (*p* < 0.001). TMB in the tumor tissues with mutated RYR3 (n = 5) was much higher than that in non-mutated RYR3 (n = 38) (*p* < 0.01). TMB in the tumor tissues with mutated TP53 (n = 12) was much higher than that in non-mutated TP53 (n = 31) (*p* < 0.01). In previous studies, the mutation status of the driver gene TP53 demonstrated the ability to predict LUAD prognosis [34, 35]. The TP53 subtype can be used as a biomarker for immune checkpoint inhibitors in LUAD [36]. All TMB values of groups with mutations in 18 genes (*p* < 0.05) were higher than those with no mutations (Table 2). It was also found that TMB was disassociated with age (Additional file 1: Figure S4A) but was associated with gender. The TMB of female patients was much lower than that of male patients (*p* < 0.05) (Additional file 1: Figure S4B). This study analyzed the differences between the landscape of LUAD’s minimally invasive adenocarcinoma (MIA) and invasive adenocarcinoma (IA). No signature was associated with infiltration (MIAs vs. IA) (Additional file 1: Figure S4C).

**Table 2:** TMB of nuclear genomes in tumor tissues between mutated and non-mutated genes

Gene name	P value*	Number of samples with somatic mutations
RYR2	< *0.001*	5
SI	< *0.01*	4
RYR3	< *0.01*	5
TP53	< *0.01*	12
USH2A	< *0.01*	3
NRXN1	< *0.01*	4
FLG	< *0.05*	3
TTN	< *0.05*	13
ZFHX4	< *0.05*	2
LRP1B	< *0.05*	5
SPTA1	< *0.05*	2
ABCA13	< *0.05*	2
RET	< *0.05*	2
ZNF831	< *0.05*	3
PCDH15	< *0.05*	2
GRIN2B	< *0.05*	2
CSMD3	< *0.05*	2
SYNE2	< *0.05*	2

*Wilcoxon signed rank test compared TMB between different groups

**Fig. 4:**
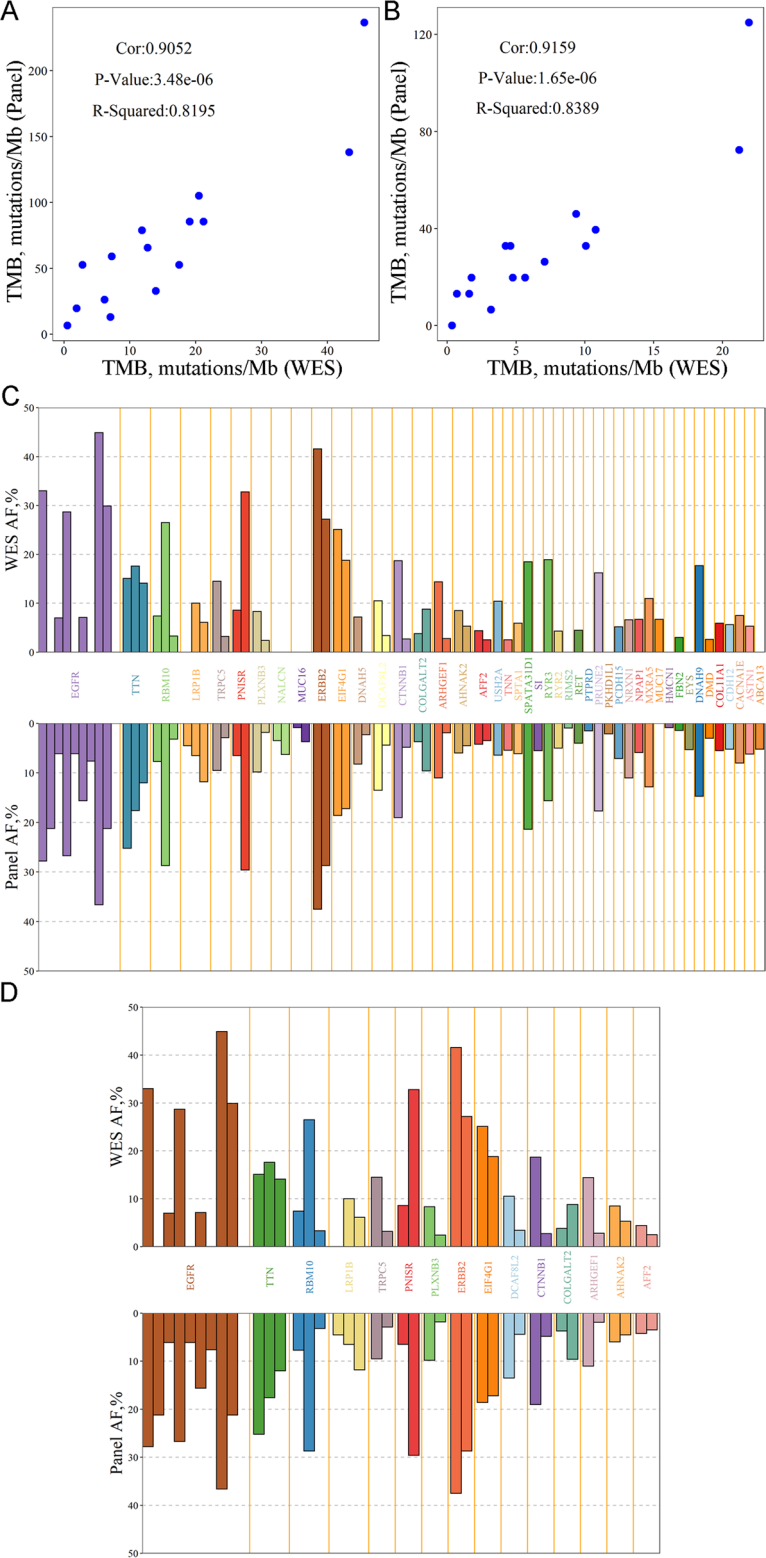
Concordance of mutation calls between targeted sequencing and WES. Concordance of TMB for all variations **A** between targeted sequencing and WES (Cor: 0.905; *p* = 3.480 × 10^–6^; R^2^ = 0.820) and **B** of protein-coding genes between targeted sequencing and WES (Cor: 0.916; *p* = 1.650 × 10^–6^; R^2^ = 0.839). Bar plot showing **C** VAFs for driver mutations in selected genes by targeted sequencing and **D** VAFs for driver mutations in selected genes by WES. For each mutation, allele frequencies were obtained by targeted sequencing and WES

Among the cohort, 15 tumor tissues and their adjacent tissue samples were performed by WES, and the correlation coefficient of TMB for all variations between WES and targeted sequencing was 0.909 (R^2^ = 0.827, *p* = 2.66 × 10^–6^) (Fig. 4A). For variations in protein-coding genes, the correlation coefficient of TMB between WES and targeted sequencing was 0.916 (R^2^ = 0.839, *p* = 1.65 × 10^–6^) (Fig. 4B). For the 15 pairs of tumor tissues and their adjacent tissue samples, 44 mutated genes were identified using targeted sequencing. Among these genes, WES identified 36 mutated genes. The VAFs for all selected mutant genes are displayed in Fig. 4C. The VAFs for the 15 mutant genes selected by WES are depicted in Fig. 4D. For each mutant gene, allele frequencies were provided for WES and targeted sequencing. All 15 mutant genes were identified by either WES or targeted sequencing. Some EGFR mutations were undetected, possibly owing to the sequencing depth. All evidence demonstrated that the targeted-captured panel aligned with expectations.

### Mutational landscape of mitochondrial genomes for LUAD tissues

**Fig. 5:**
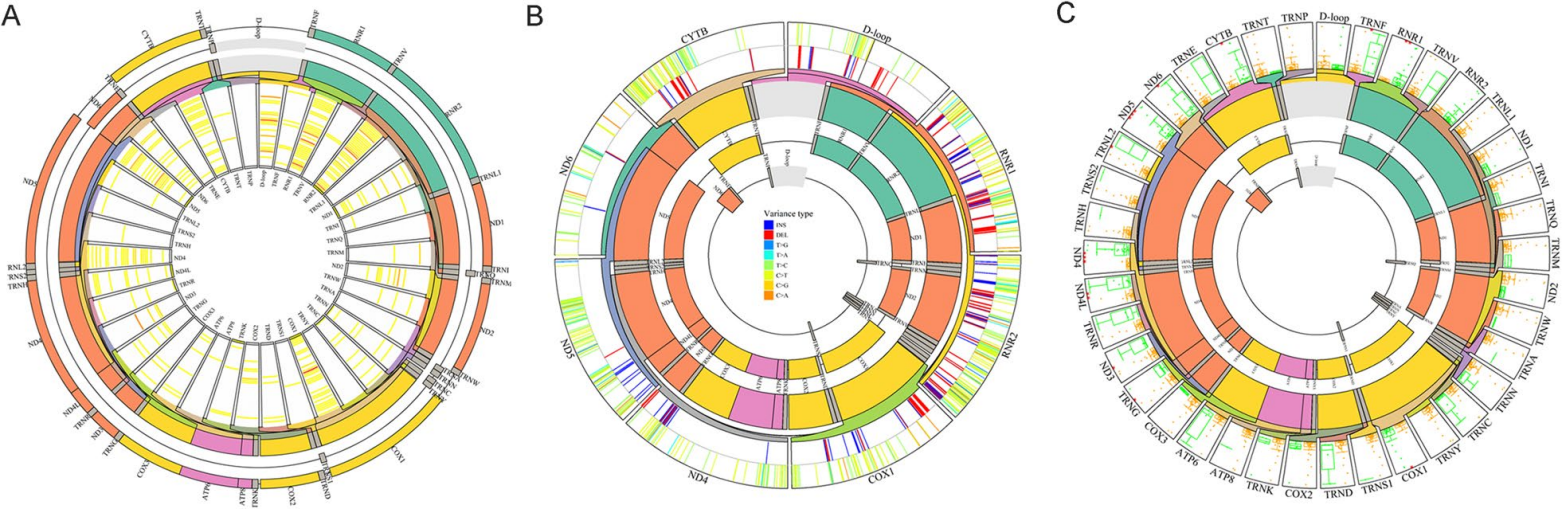
Mutation landscape of mitochondrial genome in tumor tissues of Stage IA LUAD patients. The mutation landscape of **A** all the genes in the mitochondrial genome from 43 tumor tissues of Stage IA LUAD patients, and **B** all the top five mutated genes in the mitochondrial genome from 43 tumor tissues of Stage IA LUAD patients. **C** The TMB between the tumor tissues with and without mutations in all genes in the mitochondrial genome (***p < 0.001; **p < 0.01; *p < 0.05)

This experiment subjected 43 pairs of tumor tissues and their adjacent tissue samples to captured-based mitochondrial sequencing (median depth ×3025), which would characterize the somatic mutations in mitochondrial genomes of LUAD. Then, 942 somatic mutations were identified by targeted-capture sequencing, including 122 synonymous SNVs, 757 non-synonymous SNVs, and 92 indels. Missense mutation was the leading variant classification. The non-synonymous mutation rate was significantly higher than the synonymous mutation rate. C:G>T:A (40.6%) substitution was the most frequent mutation type. All tumor tissues from 43 patients had more than one shared somatic mutation of the mitochondrial capture panel. For protein-coding genes, the top five mutated genes were cytochrome oxidase subunit I (COX1) (60.5% of patients, 26/43), NADH dehydrogenase subunit 5 (ND5) (48.8%, 21/43), cytochrome b (CYTB) (46.5%, 20/43), NADH dehydrogenase subunit 4 (ND4) (44.2%, 19/43), and NADH dehydrogenase subunit 6 (ND6) (27.9%, 12/43) (Fig. 5A). The top three mutated regions for non-protein coding in the mutational landscape of mitochondrial genomes were mitochondrially encoded 16S RNA (RNR2) (79.1%, 34/43), mitochondrially encoded 12S RNA (RNR1) (67.4%, 29/43), and the regulatory displacement loop (D-loop) region (37.2%, 16/43). The most mutated regions were RNR2, RNR1, and COX1 for synonymous and non-synonymous somatic mutations.

The locus p.N30fs in ND5 (COSM9217537) was reported in LUAD tissues from the Catalogue of Somatic Mutations in Cancer (COSMIC) database [37]. The loci p.L105P and p.A459T in ND5 (COSM9490819/ COSM1132235) and p.A59T in MT-CYB (COSM1138286) were detected in other cancers in the COSMIC database (Fig. 5B). Mutational hotspots were observed in RNR2 and RNR1 across all tumor tissue samples. Of the 13 protein-coding genes, COX1 was the most frequently mutated gene in the tumor tissue samples. Patients No. 54, No. 56, and No. 31, 47.1% (16/34), 47.1% (16/34), and 44.1% (15/34) had mutant genes or regions, respectively. In addition, TMB was associated with the mutation status of some coding genes (ND4, ND5, NADH dehydrogenase subunit 4L (ND4L), CYTB, COX1, and ND6) (Fig. 5C). ND4 harbored 57 hot spot mutations, and p.L65fs, p.L68R, p.T76M, and p.L379fs harbored more than one sample. ND5 harbored 102 hotspot mutations, and p.V147fs, p.R161LW, p.M314V, and p.H394L harbored more than one sample. Higher TMB was also related to tumors with mutated genes coding for tRNA (RNR1, tRNA-Phe (TRNF), and tRNA-Gly (TRNG)) (Fig. 5C). Overall, the TMB of groups with mutations in ten genes (*p* < 0.05) was higher than that of no mutations (Table 3). Furthermore, TMB was not associated with age or gender (Additional file 1: Figure S5A, B). There was no evidence of a differential mutation landscape between the MIAs and IA groups (Additional file 1: Figure S5C).

**Table 3:** TMB of mitochondrial genomes in tumor tissues between mutated and non-mutated genes

Gene name	P value*	Number of samples with somatic mutations
ND4	< *0.001*	19
ND5	< *0.001*	21
ND4L	< *0.01*	10
RNR1	< *0.01*	29
CYTB	< *0.05*	20
COX1	< *0.05*	25
ND3	< *0.05*	4
TRNF	< *0.05*	6
TRNG	< *0.05*	6
TRNL2	< *0.05*	2
ND6	< *0.05*	12

*Wilcoxon signed rank test compared TMB between different groups

### The concordance of mutations between cfDNA and corresponding tumor

**Fig. 6:**
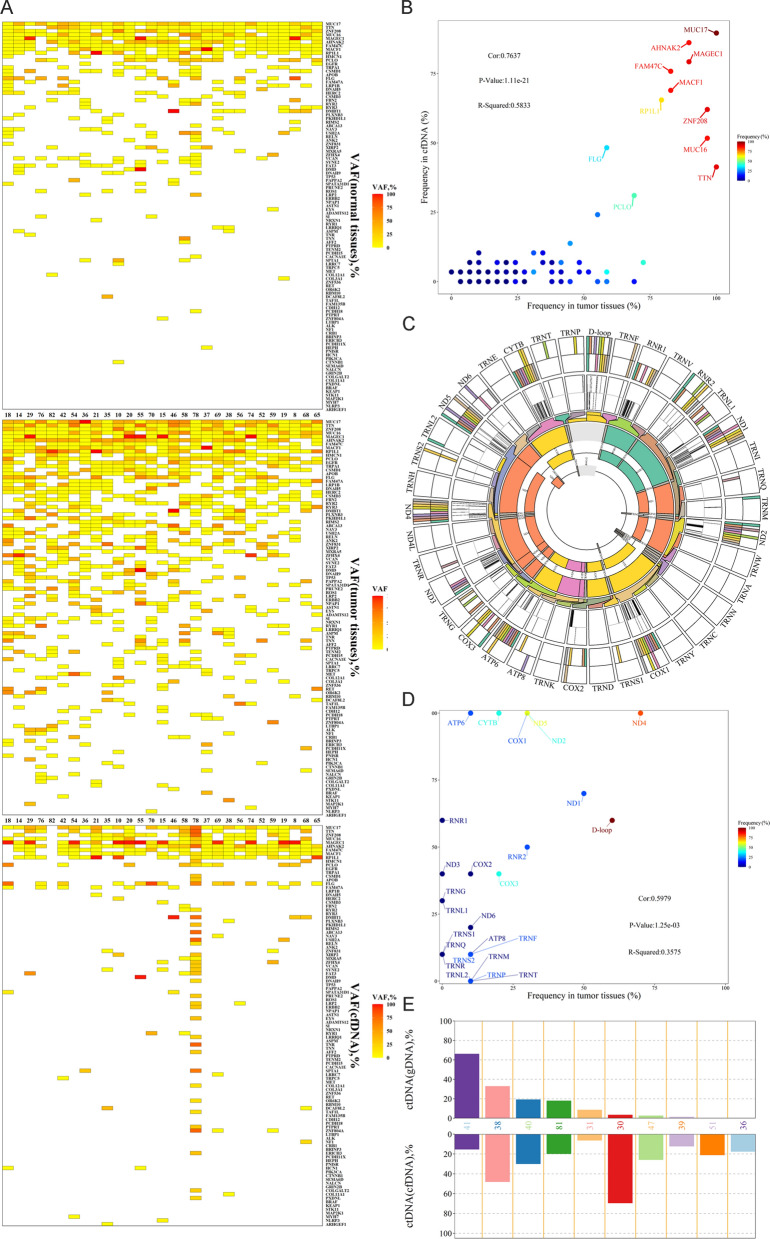
Concordance of mutation calls between solid and liquid biopsies. **A** Heatmap displayed the VAFs for mutations in selected genes of the nuclear genome. For each gene, maximum VAF was provided for 29 tumor tissues, their adjacent tissues, and plasma cfDNA samples. **B** Correlation of somatic mutation maximum VAFs of the nuclear genome in paired tumor tissue and cfDNA samples. Density estimates demonstrated a peak in mutations detected exclusively in one gene. The *p*-value was calculated using linear regression. **C** Donut chart showing the VAFs for mutations in selected genes of the mitochondrial genome. For each gene, maximum VAFs were provided for 10 tumor tissues, their adjacent tissues, and plasma cfDNA samples. The outer circle illustrated whether the 10 tumor tissues harbored mutations in relevant genes. The inner circle showed whether the ten plasma samples harbored mutations in relevant genes. **D** Correlation of somatic mutation maximum VAFs of the mitochondrial genome in paired tumor tissue and cfDNA samples. Density estimates displayed a peak in mutations detected exclusively in one gene. The *p*-value was calculated using linear regression. **E** Bar plot illustrating ctDNA fraction of mitochondrial genome in solid and liquid biopsies

In this study, 29 tumor tissues, their adjacent tissues, and plasma cfDNA samples were subjected to targeted sequencing using a custom 115 gene panel. All 29 tumor (78/115) variations were identified in the independent analysis of the cfDNA sample. Among the 29 paired samples with more than one shared somatic mutation, the hierarchy of variant allele fractions for shared mutations was highly concordant between liquid and solid biopsies (Fig. 6A). The maximum VAF of somatic mutations of genes (AHNAK2, TTN, MUC17, MUC16, MAGEC1, FAM47C, MACF1, RPL1, FLG, PCLO, and ZNF208) in tumor tissues was positively correlated with that in cfDNA (Cor = 0.759; R^2^ = 0.576; *p* = 2.65 × 10^–21^, Fig. 6B). Mutation concordance between ctDNA and matched tumor tissue was also high in bladder, prostate, and breast cancers [38–41].

This study utilized somatic mutation detection in the cfDNA and calculated the proportion of cfDNA that was tumor-derived cfDNA (ctDNA) and the ctDNA fraction to be < 1% in all 29 patients. In another lung cancer cohort, the mutant allele fraction of ctDNA detected in lung cancer patients was ~ 1%, and the ctDNA fraction for Stage I was < 1% [41], which aligned with this study. The alterations in cfDNA may have originated from blood cell proliferation and germline alterations [42, 43]. Therefore, this research focused on the concordance of mutations between cfDNA and corresponding tumors in the mitochondrial genome. Ten tumor tissues, adjacent tissues, and plasma cfDNA samples were subjected to targeted sequencing using a capture-based mitochondrial sequencing panel. The results demonstrated that 90.0% (9/10) of tumor tissue and cfDNA samples had more than one shared somatic mutation, and 60.0% (6/10) of patients had protein-altering genes with somatic mutations detected in the tumor were identified from the plasma (Fig. 6C). Among the ten paired samples with more than one shared somatic mutation of the mitochondrial genome, the hierarchy of variant allele fractions for shared mutations was highly concordant between the liquid and solid biopsies. The correlation coefficient for all somatic mutations of the mitochondrial genome in cfDNA and patient-matched tumor tissues from ten patients was 0.598, and the value of R^2^ was 0.358 (*p* = 3.84 × 10^–21^, Fig. 6D). The maximum VAFs of somatic mutations in NADH dehydrogenase subunit 1 (ND1), RNR2, and regulatory D-loop region were similar in the tumor tissue and plasma samples. In all ten patients, the ctDNA fraction ranged from 6.3 to 69.6% (Fig. 6E), which is much higher than that of the nuclear genome. It showed that the ctDNA of the mitochondrial genome was released into the blood much earlier than that of the nuclear genome because of a high copy number of the mitochondrial genome, as reported in several studies [28, 29]. The correlation coefficient for somatic mutations in mitochondrial genomes was much lower than that in nuclear genomes. However, this study recognized cell-free mtDNA as a potential tool for detection, considering its higher ctDNA fraction. The authors’ previous study indicated that the concordance of mutations between ctDNA and gDNA of the corresponding tumor was high in some mitochondria-encoding genes [27]. Due to a much higher ctDNA fraction in the mitochondrial genome, most mtDNA somatic mutations were much easier to acquire at the early stage of LUAD than in the nuclear genome.

### Mutational landscape of nuclear genomes in cfDNA of LUAD

**Fig. 7:**
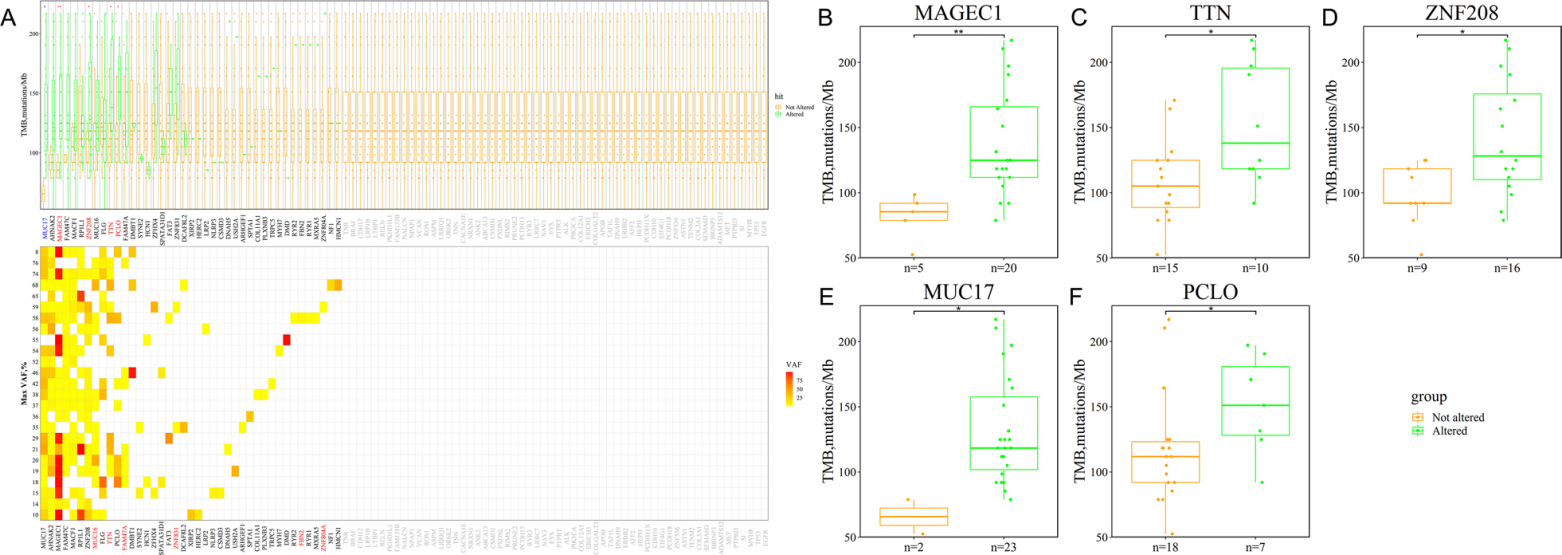
Mutation landscape of nuclear genome in cfDNA from plasma samples of Stage IA LUAD patients. **A** The mutation landscape of all the mitochondrial genome genes from 32 plasma samples of Stage IA LUAD patients. Top: the TMB between the cfDNA from plasma samples with and without mutations. Bottom: the maximum VAF for each gene in 32 plasma samples of Stage IA LUAD patients. The TMB between the cfDNA from plasma samples with and without mutations in **B** MAGEC1 (*p* < 0.01), **C** ZNF208 (*p* < 0.01), **D** PCLO (*p* < 0.01), **E** TTN (*p* < 0.01), and **F** AHNAK2 (*p* < 0.05)

Twenty-five plasma cfDNA samples from LUAD were subjected to targeted sequencing using a custom 115 gene panel to a median unique read depth of ×368. This research identified 435 somatic mutations in 55 genes by targeted-captured sequencing, including 117 synonymous SNVs, 231 non-synonymous SNVs, and 86 indels (Fig. 7A). Missense mutation was the leading variant classification. The non-synonymous mutation rate was significantly higher than the synonymous mutation rate. T:A>C:G (25.4%) substitution was the most frequent mutation type. The top five mutated genes were MUC17 (92.0%, 23/25), AHNAK2 (88.0%, 22/25), MAGEC1 (80.0%, 20/25), FAM47C (80.0%, 20/25), and MACF1(76.0%, 18/25). When the cfDNA had mutations in these genes (MAGE family member C1 (MAGEC1), TTN, ZNF208, MUC17, and piccolo presynaptic cytomatrix protein (PCLO)), the TMB was significantly higher (Fig. 7B–F). Overall, the TMB of groups with mutations in five genes (*p* < 0.05) was higher than that of groups with no mutations (Table 4). Among these 10 genes, the TMB of TTN was significantly higher in tumor tissues. Mutations in TTN harbored in cfDNA and tissues were 80.0% (10/25) and 39.5% (17/43) of patients, respectively. Mutations in TTN occurred commonly in LUAD, and a few studies reported that TTN mutations might act as a predictor for chemotherapy and immunotherapy response in LUAD patients [44, 45]. For Patient No. 58, there were 36.5% (25/115) mutated genes, including MUC17, AHNAK2, ZNF208, RP1L1, MUC16, FLG, TTN, and PCLO. For all plasma cfDNA samples from LUAD patients, TMB was not associated with age or gender (Additional file 1: Figure S6A, B). There was no significant association with infiltration (MIAs vs. IA, Additional file 1: Figure S6C).

**Table 4:** TMB of nuclear genomes in cfDNA between mutated and non-mutated genes

Gene name	P value*	Number of samples with somatic mutations
MAGEC1	< *0.01*	20
TTN	< *0.05*	10
ZNF208	< *0.05*	16
MUC17	< *0.05*	23
PCLO	< *0.05*	7

*Wilcoxon signed rank test compared TMB between different groups

### Mutational landscape of mitochondrial genomes in cfDNA of LUAD

**Fig. 8:**
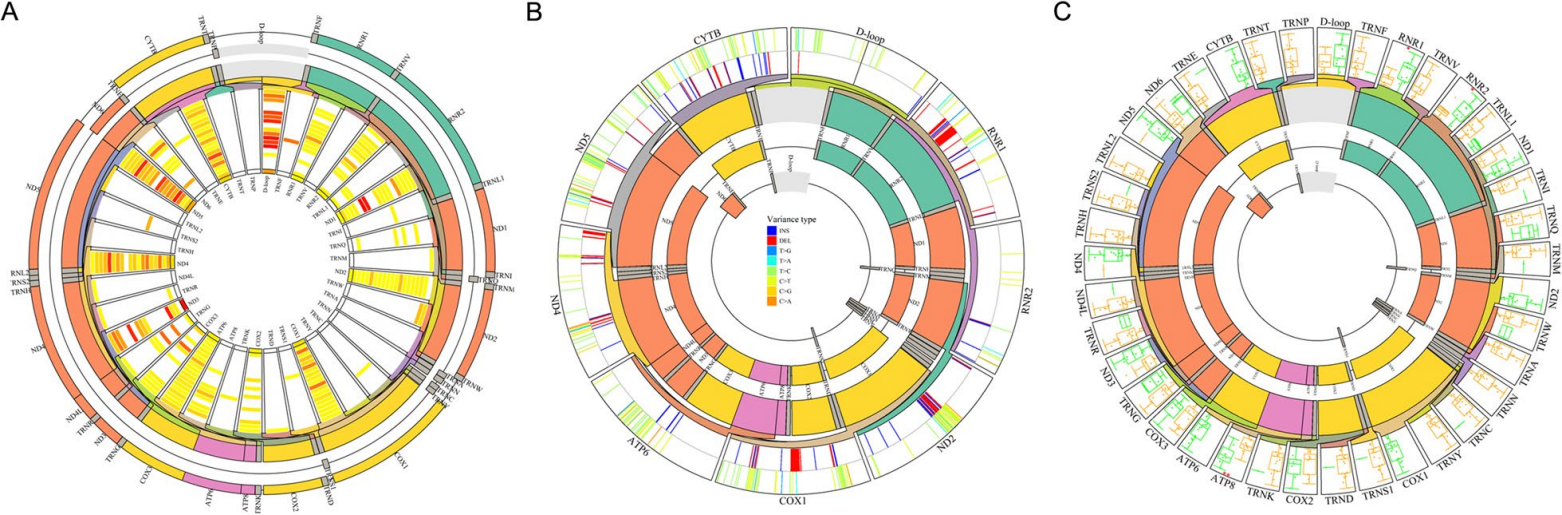
Mutation landscape of mitochondrial genome in cfDNA from plasma samples of Stage IA LUAD patients. The mutation landscape of **A** all the genes in the mitochondrial genome from 27 plasma samples of Stage IA LUAD patients, and **B** all the top five mutated genes in the mitochondrial genome from 27 plasma samples of Stage IA LUAD patients. The outer circle showed the SNVs of the top mutated genes in 27 plasma samples. The inner circle displayed the INDELs of the top mutated genes in 27 plasma samples. **C** The TMB between the tumor tissues with and without mutations in all the genes in the mitochondrial genome (**p < 0.01; *p < 0.05)

Twenty plasma cfDNA samples from LUAD were subjected to targeted sequencing using a capture-based mitochondrial sequencing panel to a median unique read depth of 2431. Overall, 30 mutated genes or regions, including 13 protein-coding genes with somatic mutations, were detected in the plasma cfDNA samples. Due to the higher sequencing depth in cfDNA than in gDNA, some low VAF mutations related to clonal hematopoiesis might not be filtered. Protein-altering somatic mutations were detected in all 20 patients. In addition, targeted-capture mitochondrial sequencing identified 647 somatic mutations, including 77 synonymous SNVs, 349 non-synonymous SNVs, and 223 indels. Missense mutation was the leading variant classification. The non-synonymous mutation rate was significantly higher than the synonymous mutation rate since the coverage of nonprotein-coding genes or regions was wider than that of the 13 protein-coding genes. T:A>C:G (34.9%) and C:G>T:A (27.3%) substitutions were the most and second-most frequent mutation types, respectively. All plasma from 20 patients had more than one shared somatic mutation of the mitochondrial capture panel. The top five mutated genes for protein-coding genes were ATP synthase F0 subunit 6 (ATP6) (100.0% of patients, 20/20), CYTB (100.0%, 20/20), COX1 (100.0%, 20/20), ND5 (95.0%, 19/20), ND4 (95.0%, 19/20), and NADH dehydrogenase subunit 2 (ND2) (95.0%, 19/20, Fig. 8A). The top three mutated regions in the mutational landscape of mitochondrial genomes for non-protein coding were RNR1 (65.0%, 13/20), RNR2 (65.0%, 13/20), and the D-loop region (65.0%, 13/20).

TMB was associated with mutations in ATP synthase F0 subunit 8 (ATP8) (*p* < 0.001), RNR1 (*p* < 0.05), and RNR2 (*p* < 0.05, Fig. 8B). ATP6 harbored 44 hot spot mutations, and the loci of p.88_89ins, p.P89fs, p.T95I, p.Q97fs, p.117_118ins, p.S119F, p.A124T, p.F128L, p.E145Q, p.L150F, p.M154V, p.V158M, p.R159H, and p.R159P, harbored more than one sample. ND5 harbored 75 hot spot mutations, and the loci of p.S104fs, p.N109K, p.G146V, p.Y159H, p.I169T, p.A267T, p.S270N, p.I29in, p.T449A, and p.F463L harbored more than one sample. ND4 harbored 48 hot spot mutations, and the loci of p.I132fs, p.T147S, p.L150fs, p.I162fs, p.I165T, p.S308N, and p.I423V harbored more than one sample (Fig. 8C). Furthermore, the average maximum VAF of the regulatory D-loop region was higher than that of other genes or regions. Most studies reported that the regulatory D-loop region was the most susceptible to either germline or somatic mutations [46, 47]. For Patient No.81, there were 42.1% (16/38) of mutated genes or regions, including ATP6, NADH dehydrogenase subunit 3 (ND3), ND4, ND5, CYTB, ND1, D-loop, RNR1, and tRNA-Met (TRNM). Patients No. 243 and No. 234 harbored more than 13 gene regions. Across the tumor tissue samples above, the protein-coding genes, including ND4, ND5, and CYTB, and the noncoding genes or regions, including the regulatory D-loop region, RNR1, and RNR2, also demonstrated high mutation frequency, which aligned with the cfDNA mutation status.

TMB was associated with the mutation status of the coding gene ATP8 (Fig. 8C), while higher TMB was also associated with tumors with mutated genes coding tRNA (RNR1 and RNR2) (Fig. 8C). Overall, the TMB of groups with mutations in three genes (*p* < 0.05) was higher than the TMB of groups with no mutations (Table 5). TMB was not associated with age or gender (Additional file 1: Figure S7A, B). TMB of the MIA group was higher than that of the IA group (Additional file 1: Figure S7C).

**Table 5:** TMB of mitochondrial genomes in tumor tissues between mutated and non-mutated genes

Gene name	P value*	Number of samples with somatic mutations
ATP8	< *0.01*	8
RNR1	< *0.05*	13
RNR2	< *0.05*	13

*Wilcoxon signed rank test compared TMB between different groups

### Plasma cfDNA diagnostic prediction for early-stage LUAD with the mutation number of hub genes in the nuclear and mitochondrial genomes

This study obtained an optimal cut-off value for plasma cfDNA mutation detection in the diagnosis of early-stage LUAD. The mutation numbers of selected genes were evaluated. Receiver operating characteristic (ROC) curve analysis explored the diagnostic potential of the selected genes in nuclear and mitochondrial genomes.

**Fig. 9:**
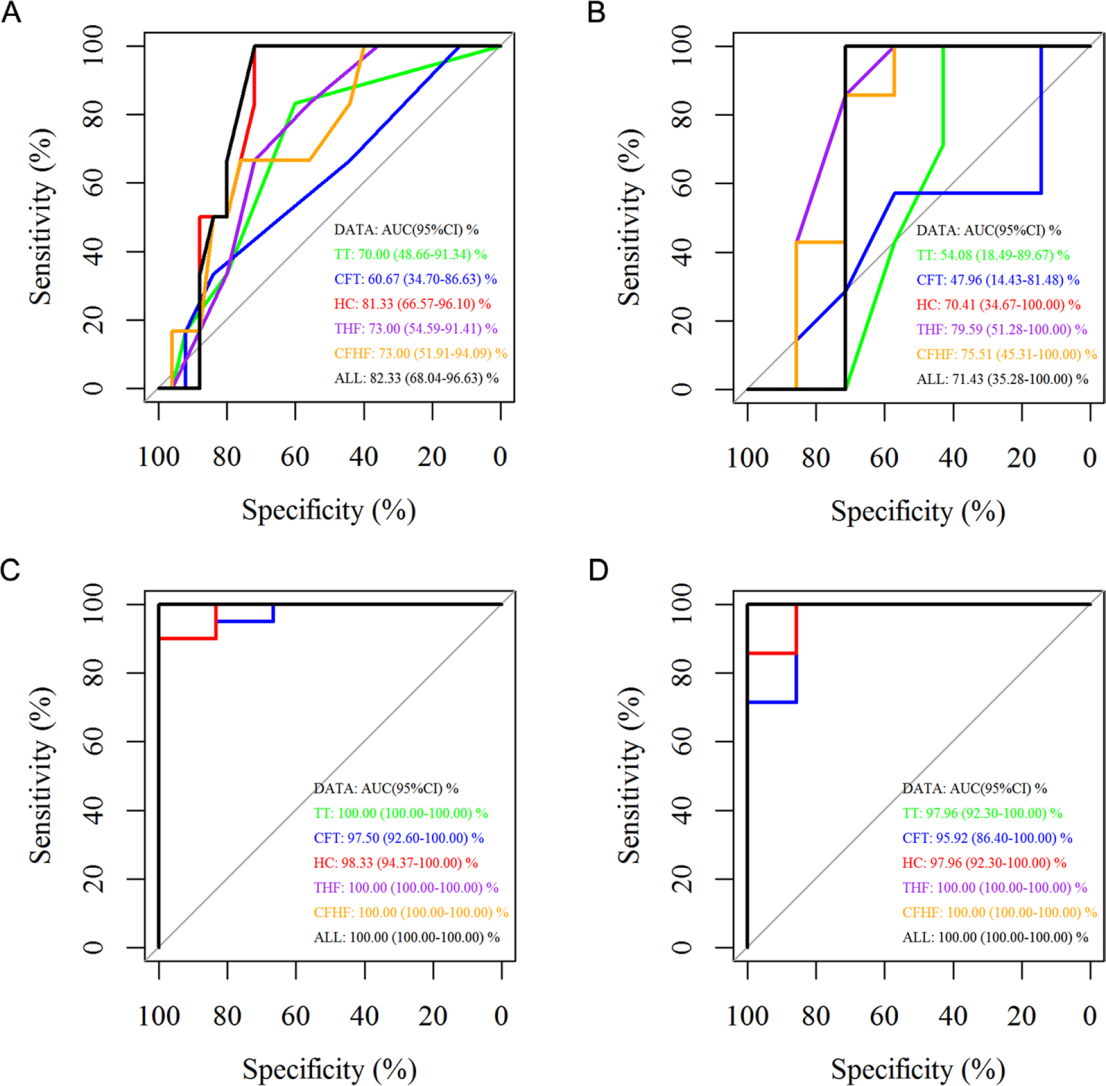
ROC analysis of the mutations in LUAD and control plasma cfDNA samples in the training data set. ROC analysis for the mutations of the nuclear genome in LUAD and control plasma cfDNA samples in the **A** training data set and **B** testing data set. ROC analysis for the mitochondrial genome mutations in LUAD and control plasma cfDNA samples in the **C** training data set and **D** testing data set. TT, the selected genes whose mutations were associated with higher TMB in tumor tissues; CFT, the selected genes that had mutations were associated with higher TMB in cfDNA of LUAD patients; HC, the selected genes whose variation frequencies in tumor tissues and cfDNA were both > 25%. THF: the top five mutated genes in tumor tissues; CFHF: the top five mutated genes in cfDNA of LUAD patients; ALL: all the above-selected genes

Furthermore, the selected genes were evaluated in a panel of the nuclear genome with 25 Stage IA LUAD patients and six healthy individuals in the training data. The selected genes included those whose mutation status was associated with higher TMB in tumor tissues or plasma cfDNA samples. The selected genes also included highly mutated genes in tumor tissues or plasma cfDNA samples and the genes’ maximum VAF had high concordance between tumor tissues and plasma cfDNA samples. The number of mutations in all selected genes in the nuclear genomic panel classified early-stage LUAD and normal individuals with a high area under curve (AUC) (82.33%, 95% CI 68.04–96.63%) in cfDNA of plasma samples (sensitivity of 100.00% and specificity of 72.00%, Fig. 9A). The number of mutations in these genes that have a maximum VAF of somatic mutations were both > 25% in tumor tissues and cfDNA of LUAD patients classified early-stage LUAD and normal individuals with high AUC (81.33%, 95% CI 66.57–96.10%) in cfDNA of plasma samples (sensitivity of 100.00% and specificity of 72.00%, Fig. 9A). The ability of plasma cfDNA diagnostic prediction for early-stage LUAD with all the groups of selected genes is illustrated in Fig. 9A and Table 6.

**Table 6:** Somatic mutations of nuclear genome in the plasma cfDNA of the training data set for the diagnosis of early-stage LUAD

	AUC (μ, 95% CI) (%)	Sensitivity (%)	Specificity (%)
TT	70.00 (48.66–91.34)	83.33	60.00
CFT	60.67 (34.70–86.63)	33.33	84.00
HC	81.33 (66.57–96.10)	100.00	72.00
THF	73.00 (54.59–91.41)	83.33	56.00
CFHF	73.00 (51.91–94.09)	66.67	76.00
ALL	82.33 (68.04–96.63)	100.00	72.00

TT: the selected genes whose mutations was associated with higher TMB in tumor tissues; CFT: the selected genes whose mutations was associated with higher TMB in cfDNA of LUAD patients; HC: the selected genes whose variation frequencies in tumor tissues and cfDNA were both > 25%; THF: the top five mutation genes in tumor tissues; CFHF: the top five mutation genes in cfDNA of LUAD patients; ALL: all the selected genes above

This research validated the plasma cfDNA diagnostic prediction for early-stage LUAD with the mutation number of hub genes in the nuclear genome. Seven Stage IA LUAD patients and seven healthy individuals were included in the testing data. The number of mutations in all selected genes in the nuclear genomic panel classified early-stage LUAD and normal individuals with a high AUC (71.43%, 95% CI 35.28–100.00%) in cfDNA of plasma samples (sensitivity of 100.00% and specificity of 71.43%) (Fig. 9B). The ability of diagnostic prediction for early-stage LUAD with plasma cfDNA in all groups of selected genes in the nuclear genome was displayed in Fig. 9B and Table 7.

**Table 7:** Somatic mutations of nuclear genome in the plasma cfDNA of the testing data set for the diagnosis of early-stage LUAD

	AUC (μ, 95% CI) (%)	Sensitivity (%)	Specificity (%)
TT	54.08 (18.49–89.67)	100.00	42.86
CFT	47.96 (14.43–81.48)	100.00	14.29
HC	70.41 (34.67–100.00)	100.00	57.15
THF	79.59 (51.28–100.00)	100.00	57.15
CFHF	75.51 (45.31–100.00)	100.00	57.15
ALL	71.43 (35.28–100.00)	100.00	71.43

This study evaluated the selected genes in a panel of the mitochondrial genome by including 20 Stage IA LUAD patients and six healthy individuals. The selection criteria of genes for plasma cfDNA diagnostic prediction for early-stage LUAD with the mutation number of hub genes in the mitochondrial genome were similar to those in the nuclear genome. The mtDNA panel of all selected genes depicted great ability to classify early-stage LUAD and normal individuals with a high AUC (100.00%, 95% CI 100.00–100.00%) in cfDNA of plasma samples (sensitivity of 100.00% and specificity of 100.00%, Fig. 9C). The mutation number of highly mutated genes also demonstrated an excellent ability to classify early-stage LUAD and normal individuals (Table 8).

**Table 8:** Somatic mutations of mitochondrial genome in the plasma cfDNA of the training data set for the diagnosis of early-stage LUAD

	AUC (μ, 95% CI) (%)	Sensitivity (%)	Specificity (%)
TT	100.00 (100.00–100.00)	100.00	100.00
CFT	97.50 (92.60–100.00)	90.00	100.00
HC	98.33 (94.37–100.00)	90.00	100.00
THF	100.00 (100.00–100.00)	100.00	100.00
CFHF	100.00 (100.00–100.00)	100.00	100.00
ALL	100.00 (100.00–100.00)	100.00	100.00

The research validated the plasma cfDNA diagnostic prediction of early-stage LUAD with the mutation number of hub genes in the mitochondrial genome by including seven Stage IA LUAD patients and seven healthy individuals in the testing data. Compared to the nuclear genome panel, the mtDNA panel revealed a better ability to classify early-stage LUAD and normal individuals with high AUC (97.15%, 95% CI 92.97–100.00%) in cfDNA of plasma samples (sensitivity of 100.00% and specificity of 100.00%) (Fig. 9D). Other groups of mitochondrial genomes also demonstrated a powerful ability to classify early-stage LUAD and normal individuals in cfDNA from plasma samples (Table 9).

**Table 9:** Somatic mutations of mitochondrial genome in the plasma cfDNA of the testing data set for the diagnosis of early-stage LUAD

	AUC (μ, 95% CI) (%)	Sensitivity (%)	Specificity (%)
TT	97.96 (92.30–100.00)	100.00	85.72
CFT	95.92 (86.40–100.00)	100.00	85.72
HC	97.96 (92.30–100.00)	100.00	85.72
THF	100.00 (100.00–100.00)	100.00	100.00
CFHF	100.00 (100.00–100.00)	100.00	100.00
ALL	100.00 (100.00–100.00)	100.00	100.00

Detection of the mutation number of selected genes in cfDNA demonstrated good diagnostic performance for early-stage LUAD. Moreover, detecting the number of somatic mutations in mitochondria can potentially be a better tool for diagnosing early-stage LUAD.

### Plasma cfDNA diagnostic prediction for early-stage LUAD with logistic regression method

This research obtained an optimal cut-off value for plasma cfDNA mutation detection in early-stage LUAD diagnosis. The Least Absolute Shrinkage and Selection Operator (LASSO) was performed. ROC curve analysis was used to explore the diagnostic ability of the selected genes in the nuclear and mitochondrial genomes.

**Fig. 10:**
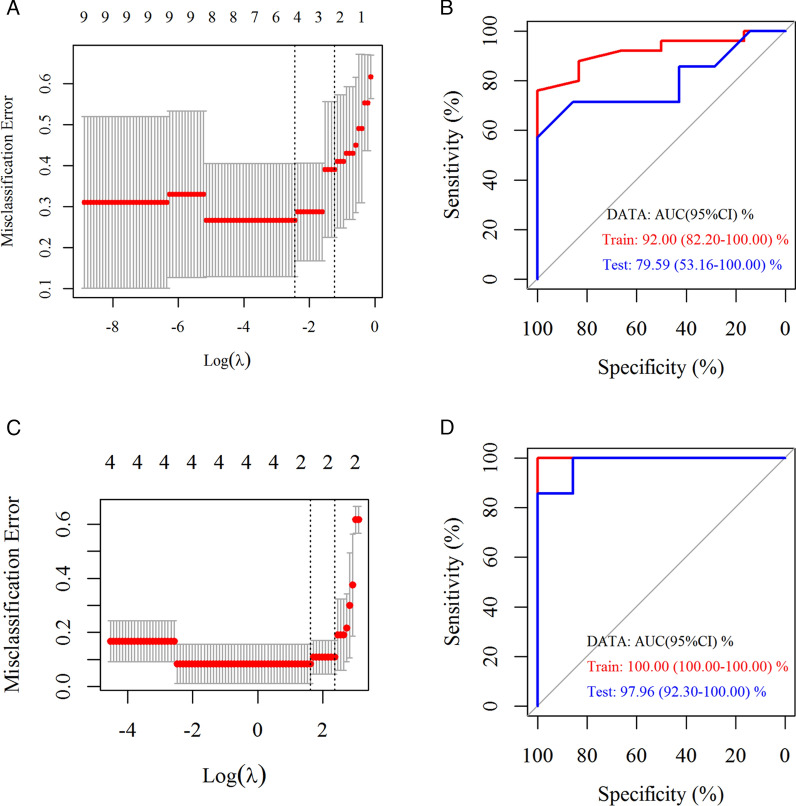
ROC analysis of the mutations in LUAD and control plasma cfDNA samples with LASSO. **A** LASSO model λ value distribution based on nuclear genome mutations in LUAD and control plasma cfDNA samples. **B** ROC analysis of the mutations of the nuclear genome in LUAD and control plasma cfDNA samples with LASSO. **C** LASSO model λ value distribution based on the mitochondrial genome mutations in LUAD and control plasma cfDNA samples. **D** ROC analysis of the mitochondrial genome mutations in LUAD and control plasma cfDNA samples with LASSO

Furthermore, the selected genes in the panel of the nuclear genome were evaluated with LASSO by including 25 Stage IA LUAD patients and six healthy individuals in the training data. LASSO analysis of the hub genes of the nuclear genome revealed that MUC17 and FAM47A were significant to LUAD diagnosis in all selected genes above (λ = 2.181) (Fig. 10A). The logistic regression method constructed a diagnostic prediction model with the two markers. The model classified early-stage LUAD and normal individuals with a high AUC (92.00%, 95% CI 82.20–100.00%) in cfDNA of plasma samples. The model yielded a sensitivity of 76.00% and specificity of 100.00% for LUAD in the training dataset of 25 LUAD and six normal samples (Fig. 10B). Plasma cfDNA diagnostic prediction was validated for early-stage LUAD with LASSO in the nuclear genome. Seven Stage IA LUAD patients and seven healthy individuals were included in the testing data. The diagnostic prediction model of LASSO for the nuclear genome classified early-stage LUAD and normal individuals with high AUC (79.59%, 95% CI 53.16–100.00%) in cfDNA of plasma samples. The model yielded a sensitivity of 71.43% and a specificity of 85.71% for LUAD (Fig. 10B).

This study included 20 Stage IA LUAD patients and six healthy individuals as the training data to evaluate the selected genes in the panel of the mitochondrial genome using LASSO. LASSO analysis of the hub genes of the mitochondrial genome showed that CYTB and RNR2 were significant to LUAD diagnosis in all selected genes (λ = 3.787) (Fig. 10C). The logistic regression method was used to construct a diagnostic prediction model using the two markers. It classified early-stage LUAD and normal individuals with a high AUC (100.00%, 95% CI 100.00–100.00%) in cfDNA of plasma samples. The model yielded a sensitivity of 100.00% and a specificity of 100.00% for LUAD (Fig. 10D). This study also validated plasma cfDNA diagnostic prediction for early-stage LUAD with LASSO in the mitochondrial genome. Seven Stage IA LUAD patients and seven healthy individuals were included in the testing data. The diagnostic prediction model of LASSO for the mitochondrial genome demonstrated that it classified early-stage LUAD and normal individuals with a high AUC (97.96%, 95% CI 92.30–100.00%) in cfDNA of plasma samples. The model yielded a sensitivity of 85.71% and a specificity of 100.00% for LUAD (Fig. 10D).

The mutation numbers for the MUC17 and FAM47A genes in the nuclear genome obtained using the LASSO diagnostic model revealed good diagnostic performance for early-stage LUAD. The mutation numbers for the CYTB and RNR2 genes in the mitochondrial genome demonstrated greater potential as a better tool for diagnosing early-stage LUAD than the selected biomarkers of the nuclear genome.

## Discussion

This study comprehensively characterized the mutated landscape of nuclear and mitochondrial genomes. Functional alterations in the nuclear genome (EGFR, TP53, TTN, and KRAS) were observed, which is largely consistent with large-scale genomic studies. In past decades, large-scale genomic studies revealed driver genes of LUAD and the most common somatic mutations harbored in the genes of TP53, KRAS, EGFR, ERBB2, MET, Ras-like without CAAX 1 (RIT1), neurofibromin 1 (NF1), kelch-like ECH associated protein 1 (KEAP1), and serine/threonine kinase 11 (STK11) [13, 15, 32]. Genomic information correlated with the diagnosis, treatment efficacy, and prognosis of LUAD.

The most common somatic mutated genes in this study cohort were verified to have clinical relevance. Patients with TTN-mutant had significantly longer overall survival (OS) than the ones with TTN-wildtype. Meanwhile, patients with TTN-mutant were found to have high immunogenicity and inflammatory tumor immune microenvironment (TIME). It suggested that TTN-mutant may be a potential predictive biomarker for LUAD patients to accept immune checkpoint inhibitors (ICIs) [44]. Several oncogenic pathways (DNA replication, mismatch repair, and spliceosome) changed noticeably in patients with TP53 mutations [48]. TP53 status was a reliable and robust immune signature for identifying early-stage LUAD patients with a high risk of unfavorable survival [49]. TCGA data demonstrated that the RYR2 mutant group lived longer than the wild group [50].

This study systematically reported the nuclear and mitochondrial mutation spectra of early-stage LUAD patients. Alterations in LUAD patients (ND4, ND5, CYTB, COX1, D-loop, RNR1, and RNR2) were observed in the mitochondrial genome. Altered energy metabolism is a common feature of cancer, and mitochondria is the primary site of energy production, which is regulated by the interplay between nuclear and mitochondrial genomes [51–53]. The human mitochondrial genome encodes 13 key proteins of four oxidative phosphorylation system (OXPHOS) complexes. It is critical for mitochondrial metabolism. Somatic mutations in the protein-coding genes of the mitochondrial genome might have effects on deregulating tumor metabolism [46].

Most studies reported that the regulatory D-loop region was the most susceptible to either germline or somatic mutations [46, 47]. In another cohort of Chinese lung cancer patients, the regulated D-loop region had a higher frequency of somatic mutations than the control region, mostly with a heterogeneous status [54]. RNR1 and hexokinase 2 (HK2) are important risk factors in hepatocellular carcinoma (HCC) patients [55]. RNR2 plays an anti-apoptotic role by avoiding deploying energy from the complete oxidation of organic compounds to inorganic wastes and could serve as a new biomarker in the diagnosis of bladder carcinoma, especially in blood circulation [56].

Most morbidity and mortality in cancer are related to late diagnosis, where clinical surgical and pharmacological treatments are less effective. Recently, liquid biopsy has emerged as a promising approach for cancer detection, monitoring of tumor progression, and response to therapy [57]. Traditional serum-based protein biomarkers (cancer antigen-125 (CA-125), cancer antigen 19-9 (CA 19-9), carcinoembryonic antigen (CEA), and prostate-specific antigen (PSA)) are commonly used for monitoring cancer progression but not for cancer diagnosis [41]. Risk factors, including genetic effects on body fluids, are still being investigated in LUAD, especially in the early stages [46]. Researchers are paying more attention to ctDNA in plasma or serum. Mutation detection in ctDNA is consistent despite intra-patient heterogeneity [38, 58]. Moreover, ctDNA can integrate somatic information from the primary tumor and multiple metastatic lesions. The intrapatient tumor heterogeneity is also similar [58].

In this study, the mutant allele fraction of ctDNA for the nuclear genome detected in LUAD patients was < 1%, which was observed in another cohort of patients with Stage I LUAD [41]. Nuclear genome alterations in cfDNA may originate from blood cell proliferation and germline alterations [42, 43]. Although ctDNA analyses have raised the possibility of direct detection of patients at an early stage of cancer, de novo identification of somatic alterations has remained a significant challenge for developing early detection approaches [59, 60]. Unlike the nuclear genome, the mitochondrial genome lacks repair mechanisms, intronic regions, and histones, making it more susceptible to damage by reactive oxygen species (ROS) and other environmental factors, leading to higher mutation frequency [61–63].

Due to the high copy number of mtDNA, this study investigated whether tumor-derived somatic mutations in the mitochondrial genome were higher than those in the nuclear genome. The correlation coefficients for all somatic mutations in the mitochondrial genome of cfDNA and patient-matched tumor tissues were lower than those in the nuclear genome. However, the ctDNA fraction of the mitochondria genome was much higher than that of the nuclear genome. This indicated that most mtDNA somatic mutations were much easier to acquire in the mitochondria genome at the early stage of LUAD than in the nuclear genome. Previous research by the authors of this study also indicated that the concordance of mutations between ctDNA and gDNA of the corresponding tumor was high in some mitochondrial encoding genes [27]. The current understanding of circulating cell-free mtDNA has the potential as a novel tumor biomarker [30].

This study comprehensively characterized the mutated landscape of nuclear and mitochondrial genomes. The number of mutations detected in related genes in plasma cfDNA samples from early-stage LUAD patients and healthy individuals was used to evaluate the diagnostic ability [64]. Functional alterations were observed in the nuclear genome (EGFR, TP53, TTN, and KRAS) and mitochondrial genomes (ND4, ND5, CYTB, COX1, D-loop, RNR1, and RNR2). For the diagnostic model of the nuclear genome, the number of mutations in these hub genes was evaluated. This study selected genes that satisfied the following criteria: (i) mutations were associated with higher TMB in tumor tissues or cfDNA of LUAD patients, (ii) the genes that had variation frequencies in tumor tissues and cfDNA were > 25%, and (iii) the top five mutated genes in tumor tissues or cfDNA of LUAD patients. The number of mutations in all selected genes in the nuclear genomic panel could classify early-stage LUAD and normal individuals with high AUC (82.33%, 95% CI 68.04–96.63%) in cfDNA of plasma samples with the training data. The diagnostic model was evaluated with the testing data, and the AUC reached 71.43% (95% CI 35.28–100.00%).

For the mitochondrial genome, the gene selection criteria for ROC were the same as described above. All selected genes in the mitochondrial genome displayed excellent ability to classify early-stage LUAD and normal individuals with a high AUC (100.00%, 95% CI 100.00–100.00%) in cfDNA of plasma samples with the training data. Similar results were observed for the testing data. Therefore, the mtDNA panel would be a better diagnostic biomarker than the nuclear genome panel with the number of mutations in selected genes. The diagnostic model analyzed by LASSO evaluated panels of nuclear and mitochondrial genomes. The mtDNA panel performed better and ensured that the diagnostic biomarkers of blood were released from tumor tissues. If the VAFs for the mutations in the tumor tissues were low, the mutations might be detected in the blood. Meanwhile, the VAFs for mutations in tumor tissues were affected by blood cell proliferation and germline alterations. The ctDNA fraction of mitochondria was much higher than that of the nuclear genome, indicating that most mtDNA somatic mutations were much easier to detect in the early stage of LUAD than in the nuclear genome. Therefore, the mitochondrial panel can classify early-stage LUAD and normal individuals in cfDNA of plasma.

This study has two limitations that should be addressed in future research. Firstly, the scope of this study was limited to one center, and all individuals were of the same race. Secondly, we expanded the sample size to obtain more credible and reliable data to accelerate clinical translation. In the future, multicenter collaboration is needed to further expand the sample size to include different regions and races to clarify the accuracy of these biomarkers for early-stage LUAD diagnosis.

## Conclusion

This study identified somatic mutations in the nuclear and mitochondrial genomes. The mutation detection of cfDNA revealed good diagnostic performance for early-stage LUAD. Moreover, somatic mutation detection in the mitochondria may be a better tool for diagnosing early-stage LUAD. The panel in the mitochondrial genome could classify primary early-stage LUAD and normal individuals in cfDNA of plasma. In the near future, we will initiate multicenter collaboration that expands the sample size from different regions and races to clarify the accuracy of these biomarkers for diagnosing early-stage LUAD.

## Supplementary Information

**Additional file 1:** **Figure S1**. TGCA early LUAD mutation cohort analyzed by SomaticSniper (**A**) Overview of TGCA Stage IA LUAD cohort mutations analyzed with the tool of SomaticSniper. (**B**) Waterfall of the top 150 mutated genes in the TCGA Stage IA LUAD cohort analyzed with the tool of SomaticSniper.<br>**Figure S2**. TGCA early LUAD mutation cohort analyzed by MuTect (**A**) Overview of TGCA Stage IA LUAD cohort mutations analyzed with the tool of MuTect. (**B**) Waterfall of the top 150 mutated genes in the TCGA Stage IA LUAD cohort analyzed with the tool of MuTect.<br>**Figure S3**. TGCA early LUAD mutation cohort analyzed by MuSE (**A**) Overview of TGCA Stage IA LUAD cohort mutations analyzed with the tool of MuSE. (**B**) Waterfall of the top 150 mutated genes in the TCGA Stage IA LUAD cohort analyzed with the tool of MuSE.<br>**Figure S4**. The TMB of nuclear and mitochondrial genomes between groups with different clinical characteristics. (**A**) The TMB of nuclear genomes in tumor tissues between the age groups of ≤60 years and >60. (**B**) The TMB of nuclear genomes in tumor tissues between the groups of male and female. (**C**) The TMB of nuclear genomes in tumor tissues between the MIAs and IA groups. (**D**) The TMB of mitochondrial genomes in tumor tissues between the age groups of ≤60 years and >60. (**E**) The TMB of mitochondrial genomes in tumor tissues between the groups of male and female. (**F**) The TMB of mitochondrial genomes in tumor tissues between the MIAs and IA groups. (**G**) The TMB of nuclear genomes in cfDNA from plasma samples between the age groups of ≤60 years and >60. (**H**) The TMB of nuclear genomes in cfDNA from plasma samples between the groups of male and female. (**I**) The TMB of nuclear genomes in cfDNA from plasma samples between the MIAs and IA groups. (**J**) The TMB of mitochondrial genomes in cfDNA from plasma samples between the age groups of ≤60 years and >60. (**K**) The TMB of mitochondrial genomes in cfDNA from plasma samples between the groups of male and female. (**L**) The TMB of mitochondrial genomes in cfDNA from plasma samples between the MIAs and IA groups.

## Data Availability

The datasets used and/or analysed during the current study are available from the corresponding author on reasonable request.

## Abbreviations

LUAD: Lung adenocarcinoma
DGCR5: DiGeorge syndrome critical region gene 5
KIF20A: Kinesin family member 20A
CLEC10A: C-type lectin domain family 10, member A
Cdh1: Cadherin 1
EpCAM: Epithelial cell adhesion molecule
EMT: Epithelial–mesenchymal transition
EGFR: Epidermal growth factor receptor
KRAS: KRAS proto-oncogene, GTPase
BRAF: B-Raf proto-oncogene, serine/threonine kinase
ERBB 2: Erb-b2 receptor tyrosine kinase 2
cfDNA: Cell free DNA
CTCs: Circulating tumor cells
TP53: Tumor protein p53
BRCA2: BRCA2 DNA repair associated
nDNA: Nuclear DNA
MT-ND1: Mitochondrially encoded NADH dehydrogenase 1
WES: Whole exome sequencing
CCDS: Consensus coding sequence
TMB: Tumor mutational burden
VAF: Variant allele frequencies
TTN: Titin
MUC16: Mucin 16, cell surface associated
CSMD3: CUB and Sushi multiple domains 3
RYR2: Ryanodine receptor 2
LRP1B: LDL receptor related protein 1B
ZFHX4: Zinc finger homeobox 4
USH2A: Usherin
FLG: Filaggrin
DMD: Dystrophin
MUC17: Mucin 17, cell surface associated
AHNAK2: AHNAK nucleoprotein 2
RBM10: RNA binding motif protein 10
AFF2: AF4/FMR2 family member 2
ARHGEF1: Rho guanine nucleotide exchange factor 1
COLGALT2: Collagen beta(1-*O*)galactosyltransferase 2
CTNNB1: Catenin beta 1
DCAF8L2: DDB1 and CUL4 associated factor 8 like 2
EIF4G1: Eukaryotic translation initiation factor 4 gamma 1
ERBB2: Erb-b2 receptor tyrosine kinase 2
PLXNB3: LRP1B, plexin B3
PNISR: PNN interacting serine and arginine rich protein
TRPC5: Transient receptor potential cation channel subfamily C member 5
NCCN: National Comprehensive Cancer Network
ALK: ALK receptor tyrosine kinase
MET: MET proto-oncogene, receptor tyrosine kinase
RET: Ret proto-oncogene
ROS1: ROS proto-oncogene 1, receptor tyrosine kinase
PIK3CA: Phosphatidylinositol-4,5-bisphosphate 3-kinase catalytic subunit alpha
NRAS: NRAS proto-oncogene, GTPase
MAP2K1: Mitogen-activated protein kinase kinase 1
COX1: Cytochrome oxidase subunit I
ND5: NADH dehydrogenase subunit 5
CYTB: Cytochrome b
ND4: NADH dehydrogenase subunit 4
ND6: NADH dehydrogenase subunit 6
RNR2: Mitochondrially encoded 16S RNA
RNR1: Mitochondrially encoded 12S RNA
D-loop: Regulatory displacement loop
COSMIC: Catalogue of Somatic Mutations in Cancer
ATP6: ATP synthase F0 subunit 6
ND2: NADH dehydrogenase subunit 2
ATP8: ATP synthase F0 subunit 8
ROC: Receiver operating characteristic
AUC: Area under curve
LASSO: Least Absolute Shrinkage and Selection Operator
RIT1: Ras like without CAAX 1
NF1: Neurofibromin 1
KEAP1: Kelch like ECH associated protein 1
STK11: Serine/threonine kinase 11
TIME: Tumor immune microenvironment
ICIs: Immune checkpoint inhibitors
OXPHOS: Oxidative phosphorylation system
HCC: Hepatocellular carcinoma
CA-125: Cancer antigen-125
CA 19-9: Cancer antigen 19-9
CEA: Carcinoembryonic antigen
PSA: Prostate-specific antigen
