# Correction: Molecular fingerprints of nuclear genome and mitochondrial genome for early diagnosis of lung adenocarcinoma

**DOI:** 10.1186/s12967-023-04128-0

**Published:** 2023-04-19

**Authors:** Yichun Xu, Yong Yang, Yichao Wang, Jun Su, Tianlong Chan, Jiajing Zhou, Yi Gong, Ke Wang, Yifeng Gu, Congmeng Zhang, Guanjin Wu, Ling Bi, Xiong Qin, Junsong Han

**Affiliations:** 1 National Engineering Research Center for Biochip at Shanghai and Shanghai Biochip Limited Corporation, No.151, Libing Road, Shanghai, 201203 China; 2 Department of Thoracic Surgery, Shanghai Pulmonary Hospital, No.241, Huaihai West Road, Shanghai, China; 3 Department of Oncology, Yueyang Hospital of Integrated Traditional Chinese and Western Medicine, Shanghai University of Traditional Chinese Medicine, No.110, Ganhe Road, Shanghai, China; 4 Department of Pathology, Shanghai Tongji Hospital, Tongji Hospital Affiliated to TongjiUniversity, Shanghai, China; 5 Institute of Interdisciplinary Integrative Medicine Research, Shanghai University of Traditional Chinese Medicine, Shanghai, China; 6 Acupuncture Anesthesia Clinical Research Institute, Yueyang Hospital of Integrated Traditional Chinese and Western Medicine, Shanghai University of Traditional Chinese Medicine, Shanghai, China

**Correction: Journal of Translational Medicine (2023) 21:250** **https://doi.org/10.1186/s12967-023-04099-2**

Following publication of the original article [1], we have been notified that author Ling Bi was not mentioned as one of the corresponding authors. This should be mentioned as follows:

Ling Bi^3,5*^
