# Supplementary material for: Molecular fingerprints of nuclear genome and mitochondrial genome for early diagnosis of lung adenocarcinoma
Source: J Transl Med. 2023 Apr 10;21:250. doi: 10.1186/s12967-023-04099-2 (PMC10084603; doi:10.1186/s12967-023-04099-2)
Supplement: Supplementary file 1 — Additional file 1: Figure S1. TGCA early LUAD mutation cohort analyzed by SomaticSniper (A) Overview of TGCA Stage IA LUAD cohort mutations analyzed with the tool of SomaticSniper. (B) Waterfall of the top 150 mutated genes in the TCGA Stage IA LUAD cohort analyzed with the tool of SomaticSniper.Figure S2. TGCA early LUAD mutation cohort analyzed by MuTect (A) Overview of TGCA Stage IA LUAD cohort mutations analyzed with the tool of MuTect. (B) Waterfall of the top 150 mutated genes in the TCGA Stage IA LUAD cohort analyzed with the tool of MuTect.Figure S3. TGCA early LUAD mutation cohort analyzed by MuSE (A) Overview of TGCA Stage IA LUAD cohort mutations analyzed with the tool of MuSE. (B) Waterfall of the top 150 mutated genes in the TCGA Stage IA LUAD cohort analyzed with the tool of MuSE.Figure S4. The TMB of nuclear and mitochondrial genomes between groups with different clinical characteristics. (A) The TMB of nuclear genomes in tumor tissues between the age groups of ≤60 years and >60. (B) The TMB of nuclear genomes in tumor tissues between the groups of male and female. (C) The TMB of nuclear genomes in tumor tissues between the MIAs and IA groups. (D) The TMB of mitochondrial genomes in tumor tissues between the age groups of ≤60 years and >60. (E) The TMB of mitochondrial genomes in tumor tissues between the groups of male and female. (F) The TMB of mitochondrial genomes in tumor tissues between the MIAs and IA groups. (G) The TMB of nuclear genomes in cfDNA from plasma samples between the age groups of ≤60 years and >60. (H) The TMB of nuclear genomes in cfDNA from plasma samples between the groups of male and female. (I) The TMB of nuclear genomes in cfDNA from plasma samples between the MIAs and IA groups. (J) The TMB of mitochondrial genomes in cfDNA from plasma samples between the age groups of ≤60 years and >60. (K) The TMB of mitochondrial genomes in cfDNA from plasma samples between the groups of male and female. (L) The TMB of mitoch [file 12967_2023_4099_MOESM1_ESM.docx]

**Figures**

**Figure S1**

**
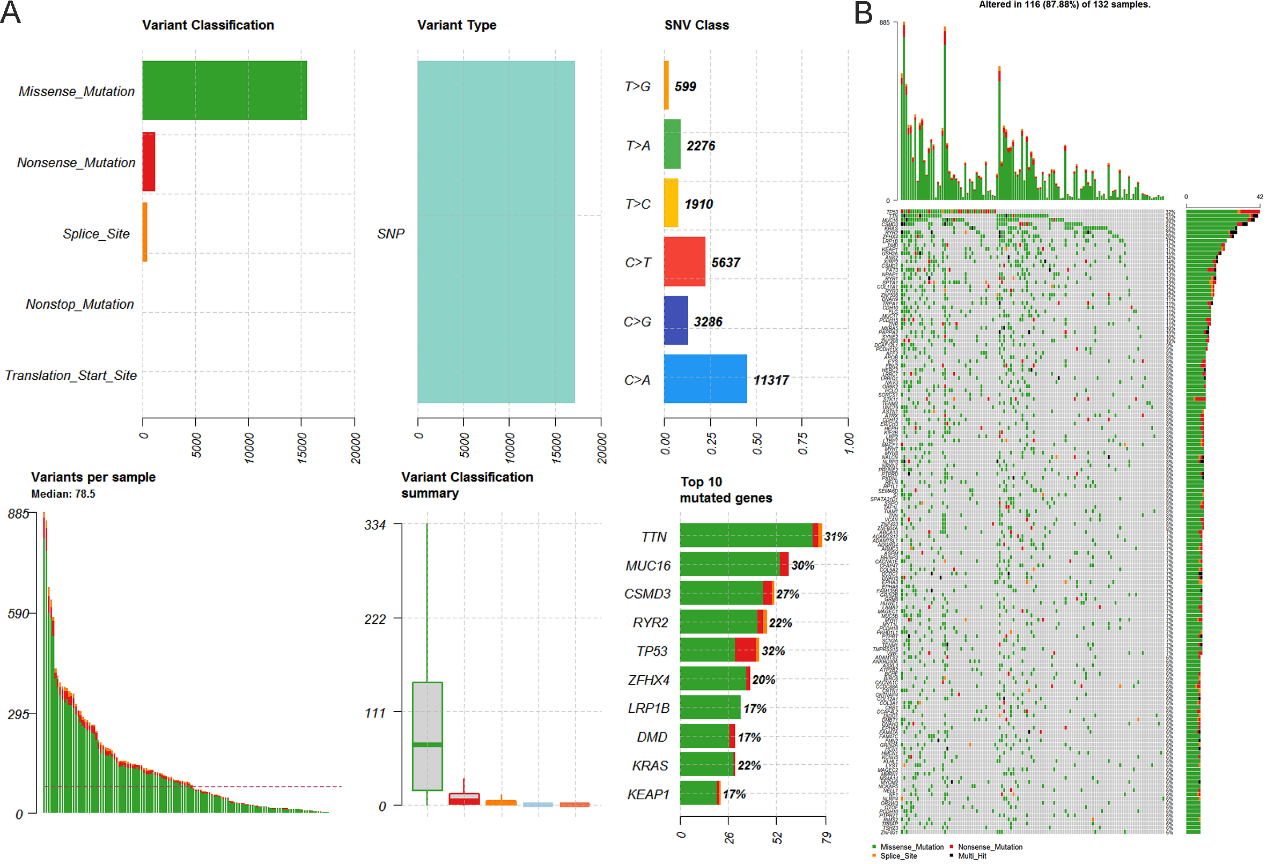
**

**Figure S2**

**
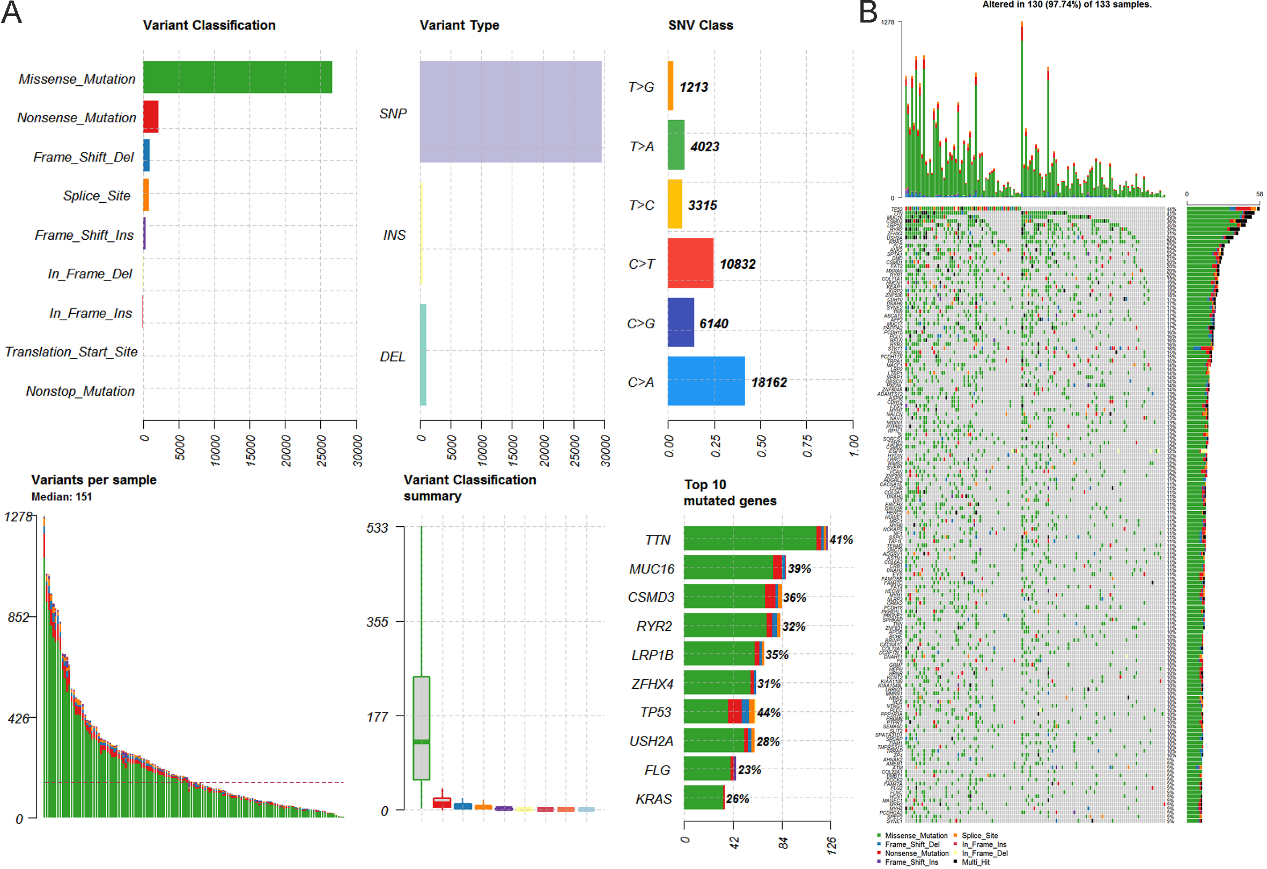
**

**Figure S3**

**
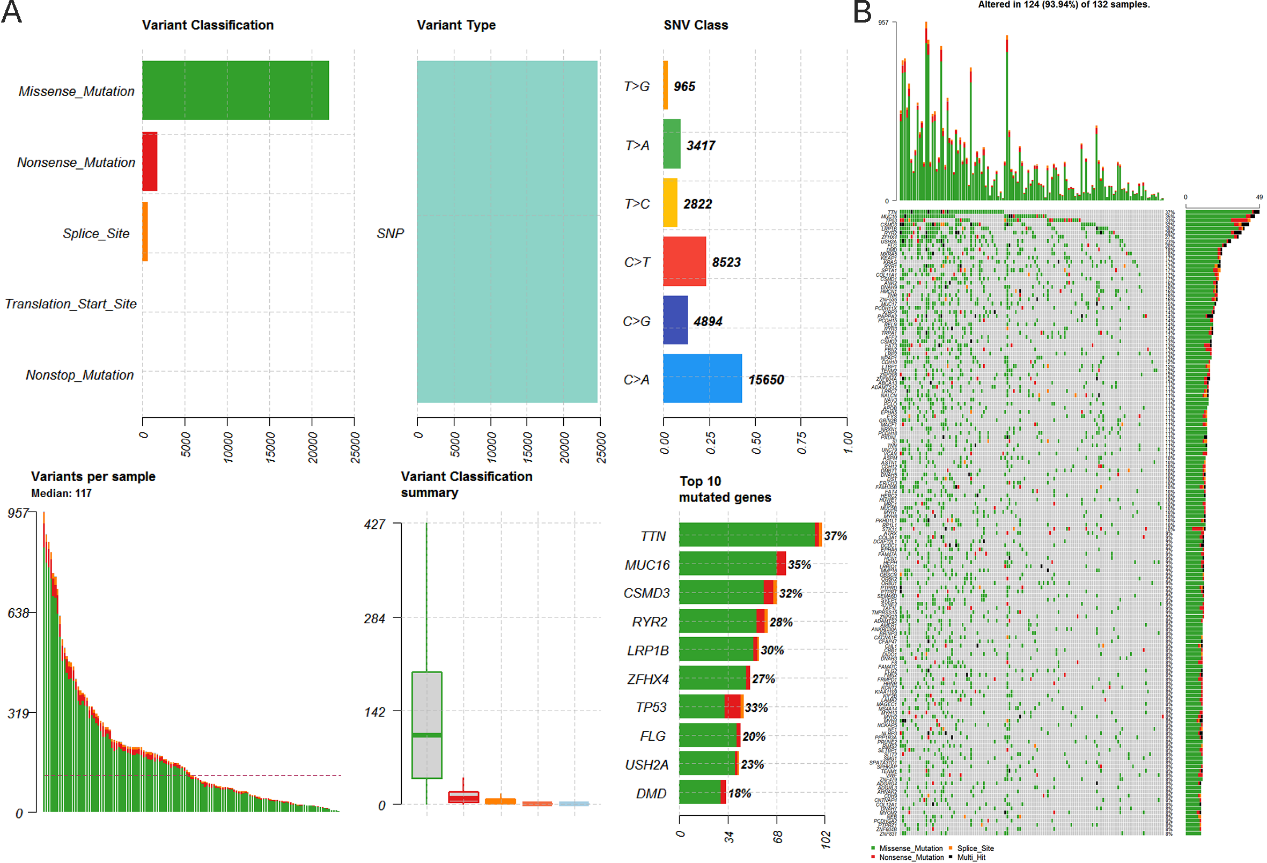
**

**Figure S4**

**
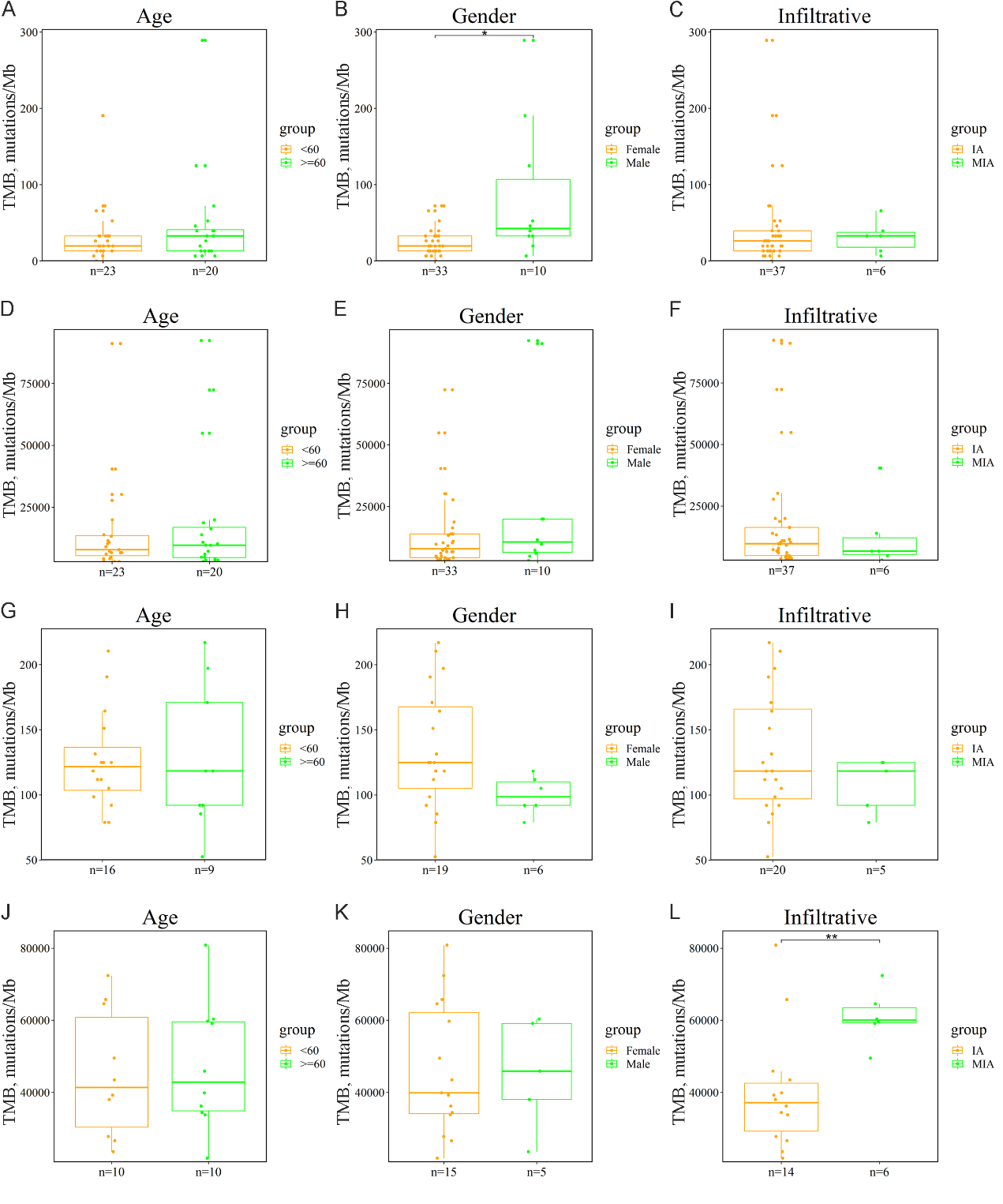
**

**Tables**

**Table S1 Genes involved in the panel of nuclear genome**

| **Symbol** | **Gene_ID** | **Description** |
| --- | --- | --- |
| ABCA13 | 154664 | ATP binding cassette subfamily A member 13 |
| ADAMTS12 | 81792 | ADAM metallopeptidase with thrombospondin type 1 motif 12 |
| AFF2 | 2334 | AF4/FMR2 family member 2 |
| AHNAK2 | 113146 | AHNAK nucleoprotein 2 |
| ALK | 238 | ALK receptor tyrosine kinase |
| ANK2 | 287 | ankyrin 2 |
| APOB | 338 | apolipoprotein B |
| ARHGEF1 | 9138 | Rho guanine nucleotide exchange factor 1 |
| ASPM | 259266 | assembly factor for spindle microtubules |
| ASTN1 | 460 | astrotactin 1 |
| BRAF | 673 | B-Raf proto-oncogene, serine/threonine kinase |
| BRINP3 | 339479 | BMP/retinoic acid inducible neural specific 3 |
| CACNA1E | 777 | calcium voltage-gated channel subunit alpha1 E |
| CDH10 | 1008 | cadherin 10 |
| CDH12 | 1010 | cadherin 12 |
| COL11A1 | 1301 | collagen type XI alpha 1 chain |
| COL12A1 | 1303 | collagen type XII alpha 1 chain |
| COL3A1 | 1281 | collagen type III alpha 1 chain |
| COLGALT2 | 23127 | collagen beta(1-O)galactosyltransferase 2 |
| CRB1 | 23418 | crumbs cell polarity complex component 1 |
| CSMD1 | 64478 | CUB and Sushi multiple domains 1 |
| CSMD3 | 114788 | CUB and Sushi multiple domains 3 |
| CTNNB1 | 1499 | catenin beta 1 |
| DCAF12L1 | 139170 | DDB1 and CUL4 associated factor 12 like 1 |
| DCAF8L2 | 347442 | DDB1 and CUL4 associated factor 8 like 2 |
| DMBT1 | 1755 | deleted in malignant brain tumors 1 |
| DMD | 1756 | dystrophin |
| DNAH5 | 1767 | dynein axonemal heavy chain 5 |
| DNAH9 | 1770 | dynein axonemal heavy chain 9 |
| EGFR | 1956 | epidermal growth factor receptor |
| EIF4G1 | 1981 | eukaryotic translation initiation factor 4 gamma 1 |
| ERBB2 | 2064 | erb-b2 receptor tyrosine kinase 2 |
| ERICH3 | 127254 | glutamate rich 3 |
| EYS | 346007 | eyes shut homolog |
| FAM135B | 51059 | family with sequence similarity 135 member B |
| FAM47A | 158724 | family with sequence similarity 47 member A |
| FAM47C | 442444 | family with sequence similarity 47 member C |
| FAT3 | 120114 | FAT atypical cadherin 3 |
| FBN2 | 2201 | fibrillin 2 |
| FLG | 2312 | filaggrin |
| GRIN2B | 2904 | glutamate ionotropic receptor NMDA type subunit 2B |
| HCN1 | 348980 | hyperpolarization activated cyclic nucleotide gated potassium channel 1 |
| HEPH | 9843 | hephaestin |
| HERC2 | 8924 | HECT and RLD domain containing E3 ubiquitin protein ligase 2 |
| HMCN1 | 83872 | hemicentin 1 |
| KEAP1 | 9817 | kelch like ECH associated protein 1 |
| KRAS | 3845 | KRAS proto-oncogene, GTPase |
| LRP1B | 53353 | LDL receptor related protein 1B |
| LRP2 | 4036 | LDL receptor related protein 2 |
| LRRC7 | 57554 | leucine rich repeat containing 7 |
| LRRIQ1 | 84125 | leucine rich repeats and IQ motif containing 1 |
| LTBP1 | 4052 | latent transforming growth factor beta binding protein 1 |
| MACF1 | 23499 | microtubule actin crosslinking factor 1 |
| MAGEC1 | 9947 | MAGE family member C1 |
| MAP2K1 | 5604 | mitogen-activated protein kinase kinase 1 |
| MET | 4233 | MET proto-oncogene, receptor tyrosine kinase |
| MUC16 | 94025 | mucin 16, cell surface associated |
| MUC17 | 140453 | mucin 17, cell surface associated |
| MXRA5 | 25878 | matrix remodeling associated 5 |
| MYH7 | 4625 | myosin heavy chain 7 |
| MYH8 | 4626 | myosin heavy chain 8 |
| NALCN | 259232 | sodium leak channel, non-selective |
| NAV3 | 89795 | neuron navigator 3 |
| NF1 | 4763 | neurofibromin 1 |
| NLRP3 | 114548 | NLR family pyrin domain containing 3 |
| NPAP1 | 23742 | nuclear pore associated protein 1 |
| NRAS | 4893 | NRAS proto-oncogene, GTPase |
| NRXN1 | 9378 | neurexin 1 |
| OR6K2 | 81448 | olfactory receptor family 6 subfamily K member 2 |
| PAPPA2 | 60676 | pappalysin 2 |
| PCDH11X | 27328 | protocadherin 11 X-linked |
| PCDH15 | 65217 | protocadherin related 15 |
| PCDH18 | 54510 | protocadherin 18 |
| PCLO | 27445 | piccolo presynaptic cytomatrix protein |
| PIK3CA | 5290 | phosphatidylinositol-4,5-bisphosphate 3-kinase catalytic subunit alpha |
| PKHD1L1 | 93035 | PKHD1 like 1 |
| PLXNB3 | 5365 | plexin B3 |
| PNISR | 25957 | PNN interacting serine and arginine rich protein |
| PRUNE2 | 158471 | prune homolog 2 with BCH domain |
| PTPRD | 5789 | protein tyrosine phosphatase receptor type D |
| PTPRT | 11122 | protein tyrosine phosphatase receptor type T |
| PXDNL | 137902 | peroxidasin like |
| RBM10 | 8241 | RNA binding motif protein 10 |
| RELN | 5649 | reelin |
| RET | 5979 | ret proto-oncogene |
| RIMS2 | 9699 | regulating synaptic membrane exocytosis 2 |
| ROS1 | 6098 | ROS proto-oncogene 1, receptor tyrosine kinase |
| RP1L1 | 94137 | RP1 like 1 |
| RYR1 | 6261 | ryanodine receptor 1 |
| RYR2 | 6262 | ryanodine receptor 2 |
| RYR3 | 6263 | ryanodine receptor 3 |
| SEMA6D | 80031 | semaphorin 6D |
| SI | 6476 | sucrase-isomaltase |
| SPATA31D1 | 389763 | SPATA31 subfamily D member 1 |
| SPTA1 | 6708 | spectrin alpha, erythrocytic 1 |
| STK11 | 6794 | serine/threonine kinase 11 |
| SYNE2 | 23224 | spectrin repeat containing nuclear envelope protein 2 |
| TAF1L | 138474 | TATA-box binding protein associated factor 1 like |
| TENM2 | 57451 | teneurin transmembrane protein 2 |
| TMPRSS15 | 5651 | transmembrane serine protease 15 |
| TNN | 63923 | tenascin N |
| TNR | 7143 | tenascin R |
| TP53 | 7157 | tumor protein p53 |
| TRPA1 | 8989 | transient receptor potential cation channel subfamily A member 1 |
| TRPC5 | 7224 | transient receptor potential cation channel subfamily C member 5 |
| TTN | 7273 | titin |
| UNC79 | 57578 | unc-79 homolog, NALCN channel complex subunit |
| USH2A | 7399 | usherin |
| VCAN | 1462 | versican |
| XIRP2 | 129446 | xin actin binding repeat containing 2 |
| ZFHX4 | 79776 | zinc finger homeobox 4 |
| ZNF208 | 7757 | zinc finger protein 208 |
| ZNF536 | 9745 | zinc finger protein 536 |
| ZNF804A | 91752 | zinc finger protein 804A |
| ZNF831 | 128611 | zinc finger protein 831 |
